# Comparative mitochondrial genomics in Nematoda reveal astonishing variation in compositional biases and substitution rates indicative of multi-level selection

**DOI:** 10.1186/s12864-024-10500-1

**Published:** 2024-06-18

**Authors:** Eli M. S. Gendron, Xue Qing, Joseph L. Sevigny, Hongmei Li, Zhiyin Liu, Mark Blaxter, Thomas O. Powers, W. Kelly Thomas, Dorota L. Porazinska

**Affiliations:** 1 Department of Entomology and Nematology, University of Florida, Gainesville, FL USA; 2 Department of Plant Pathology, Nanjing Agricultural University, Nanjing, China; 3 Molecular, Cellular, and Biomedical Sciences, University of New Hampshire, Durham, NH USA; 4 Hubbard Center for Genome Studies, University of New Hampshire, Durham, NH USA; 5 Wellcome Sanger Institute, Cambridge, UK; 6 Department of Plant Pathology, University of Nebraska, Lincoln, NE USA

**Keywords:** Evolution, GC skew, Genome size, Mitochondria, Nematode, Protein coding genes, Selection, Traits

## Abstract

**Background:**

Nematodes are the most abundant and diverse metazoans on Earth, and are known to significantly affect ecosystem functioning. A better understanding of their biology and ecology, including potential adaptations to diverse habitats and lifestyles, is key to understanding their response to global change scenarios. Mitochondrial genomes offer high species level characterization, low cost of sequencing, and an ease of data handling that can provide insights into nematode evolutionary pressures.

**Results:**

Generally, nematode mitochondrial genomes exhibited similar structural characteristics (e.g., gene size and GC content), but displayed remarkable variability around these general patterns. Compositional strand biases showed strong codon position specific G skews and relationships with nematode life traits (especially parasitic feeding habits) equal to or greater than with predicted phylogeny. On average, nematode mitochondrial genomes showed low non-synonymous substitution rates, but also high clade specific deviations from these means. Despite the presence of significant mutational saturation, non-synonymous (dN) and synonymous (dS) substitution rates could still be significantly explained by feeding habit and/or habitat. Low ratios of dN:dS rates, particularly associated with the parasitic lifestyles, suggested the presence of strong purifying selection.

**Conclusions:**

Nematode mitochondrial genomes demonstrated a capacity to accumulate diversity in composition, structure, and content while still maintaining functional genes. Moreover, they demonstrated a capacity for rapid evolutionary change pointing to a potential interaction between multi-level selection pressures and rapid evolution. In conclusion, this study helps establish a background for our understanding of the potential evolutionary pressures shaping nematode mitochondrial genomes, while outlining likely routes of future inquiry.

**Supplementary Information:**

The online version contains supplementary material available at 10.1186/s12864-024-10500-1.

## Background

Nematodes are among the most ubiquitous, abundant, and diverse animals on Earth [1]. They are adapted to every habitat, ranging from the deepest oceans to the driest deserts [2, 3]. Through their diversity of feeding traits (i.e., bacterivores, fungivores, plant and animal parasites, omnivores, and predators) [4, 5] and positioning across most trophic levels within food webs, nematodes are significant contributors to many ecosystem functions (e.g., primary productivity, decomposition, and overall nutrient cycling) [6–10]. Unfortunately, out of the estimated 1 – 10 million species less than 30,000 have been described resulting in substantial gaps in the knowledge of nematode biology, ecology, and evolution [1].

The current understanding of nematode evolutionary relationships is derived from analyses of three main molecular approaches: ribosomal genes (primarily 18S rRNA) [11–15], genomes/transcriptomes [16, 17], and mitochondrial genes [18, 19]. These studies generally recognize three main lineages (i.e., Enoplia, Dorylaimia, and Chromadoria), with Enoplia recently considered to be sister to the rest of Nematoda [17, 20]. While the 18S rRNA based phylogeny includes the largest taxon representation (2,700 sequences across the entire phylum), it lacks resolving power to discriminate between closely related taxa [15, 19]. While genome-based phylogenies (both nuclear and mitochondrial) have a higher potential to provide these detailed insights, they are significantly curtailed by the current state of limited taxon sampling (i.e., ~ 300 nuclear genomes/transcriptomes and ~ 250 mitochondrial genomes) [17, 21, 22], which are also heavily biased towards animal and plant-parasites.

Although all evolutionary frameworks have been generally congruent, significant discrepancies between nuclear and mitochondrial data have been observed for some clades (e.g., unsupported monophylies of Tylenchina and Spirurina and major animal and plant parasites, in mitochondrial based phylogenies) [23–26]. Some of the hypothesized factors include high replication and evolutionary rates in mitochondrial sequences, present throughout metazoans [27, 28], resulting in substitution saturation occluding accurate recovery of relationships, especially at the deeper tree branches [29]. While nematode mitochondrial data may produce phylogenies inconsistent with nuclear data, they can provide a lens into evolutionary pressures and mechanisms driving nematode diversity, as they have in other taxa (e.g., *Drosophila* sp.) [30, 31].

The mitochondrial genomes of many metazoans (e.g., Arthropoda, Cnidaria, Porifera, Mollusca) have been well characterized [20, 31–35], but those of nematodes have received less attention. There are currently ~ 250 published full mitochondrial genomes, with the great majority representing a single order of Rhabditida within Chromadoria [19] (Table 1, SI Table 1). Basal orders to Chromadoria (e.g., Chromadorida, Desmoscolecida, and Monhysterida) as well as orders closer to the root of the nematode tree (e.g., Enoplida, Triplonchida, and Mononchida) have not yet been sequenced. Moreover, nematode genomes have been dominated by animal (~ 56%) and plant (~ 21%) parasites [22], so to allow for meaningful comparative analyses at the phylum-wide level, mitochondrial genome coverage needs to be expanded upon by increasing taxon sampling across these taxonomic and trait gaps.

**Table 1:** Nematoda mitochondrial genomes summary

Class	Subclass	Order	Suborder	Infraorder	Genomes Generated for this Study	Publicly available Genomes	Total Collected Genomes	
Chromadorea	Chromadoria	Rhabditida	Rhabditina	Rhabditomorpha	1	83	84	
				Diplogasteromorpha	6	3	9	
			Spirurina	Ascaridomorpha		33	33	
				Spiruromorpha		23	23	
				Oxyuridomorpha		7	7	
				Rhigonematomorpha		1	1	
				Gnathostomatomorpha		2	2	
				Spirurina *incertae sedis*		2	2	
			Tylenchina	Tylenchomorpha	19	20	39	
				Panagrolaimomorpha	8	9	17	
				Cephalobomorpha	1	1	2	
		Plectida	Plectina		1	1	2	
Enoplea	Dorylaimia	Mermithida	Mermithina			7	7	
		Trichinellida				16	16	
		Dorylaimida	Dorylaimina		7	5	12	
			Nygolaimina		1		1	
	Enoplia	Enoplida	Alaimina		1		1	
			Trefusiina		2		2	
		Triplonchida	Tobrilina		1		1	

Summary of genomes used in the study, including both publicly sourced genomes and genomes sequenced and assembled for this study. Genome counts are organized by current standing assigned taxonomic ranks down to the infraorder level

Generally, mitochondrial genomes of nematodes are similar to those of other animals. They usually consist of a single circular chromosome (but see *Globodera pallida* for an example of a multipartite mitochondrial genome) [36] that range in size from 12.6 kb (*Xiphinema americanum*) [37] to 26 kb (*Romanomermis culicivorax*) [38]. They tend to contain 22 transfer RNA genes (tRNAs) and 2 ribosomal RNA genes (rRNA) [19]. However, unlike other metazoans, nematode mitochondrial genomes contain only 12 protein coding genes (PCGs) with the *atp8* gene usually missing [19]. The overall genome size variation of Nematoda falls within the reported range of Metazoa, especially towards the lower size limit (13 – 22 kb) [20, 34, 39, 40]. Similar to other metazoans, the genome size variation has been attributed to hypervariable noncoding, often repetitive, regions. However, nematode mitochondrial genomes are some of the smallest of the metazoans, with the narrowest variation in size of all animal genomes [34, 40, 41]. Like other metazoans, nematode mitogenomes predominately display high AT nucleotide richness ranging from 43% (*Panogrolaimus rigidus*) to 87% *(Meloidogyne exigua).* In contrast with other metazoans, within the coding strand of their mitochondrial genes, nematodes display a wide range of compositional skews. Nematoda and Platyhelminthes have some of the highest GC and lowest AT skews of the metazoans. These skews have been often cited as evidence of high replication rates resulting in the deamination of cytosine leading to thymine/uracil accumulation and an increased guanine ratio [34, 42, 43]. The ubiquitous and diverse nature, not only of nematode lifestyles, but also mitochondrial genomes provides us with a unique opportunity to begin to link life histories and niche preferences with genomic characteristics. This linkage can provide insights into how niche selection pressures influence the evolution of nematode genomes as well as a rationale for the unique characteristics of Nematoda compared to other metazoans. However, since most studies of nematode mitochondrial genomes have been restricted to either single genomes or a handful of similar genomes, a systematic comparison across the entire phylum is still missing from the literature.

Here, we aimed to provide a foundation of knowledge on the characteristics of nematode mitochondrial genomes and provide context on the potential role of niche selection pressures on the development and evolution of mitochondrial genomes. We examined 261 mitochondrial genomes, spanning the entire Nematoda phylum (214 published in NCBI and 47 recently sequenced) (Table 1, SI Table 1), to systematically describe their characteristics. Specifically, we hypothesized that life traits associated with feeding habits, habitat, and reproduction strategies, instead of direct phylogenetic relationships, would have the most impact on the composition and structure of these genomes. We utilized comparative mitochondrial metagenomics to focus on genome size variation, nucleotide composition, strand compositional bias, and rates of synonymous (dS) and non-synonymous (dN) nucleotide substitutions to compare how these factors were associated with the measured trait characteristics.

## Methods

### Data acquisition and mitochondrial genome sequencing

We collated a total of 261 ‘complete’ nematode mitochondrial genomes (‘complete’ defined as possessing 12 PCG sequences) representing the major clades of the phylum Nematoda (Table 1, SI Table 1). The genomes covered both shallow and deep branches of phylogenetic relationships allowing for wider (e.g., phylum level) and finer (e.g., family and genus level) comparisons. Out of these 261 genomes, 214 genomes were sourced from the NCBI GenBank database, 27 genomes were sourced from a previous publication [22], and 20 were produced specifically for this study (SI Table 1). The genomes of the 20 species were produced at the Nanjing Agricultural University, China. Nematodes were extracted with modified Whitehead trays [44]. Nematodes were extracted, placed onto Petri dishes, identified morphologically, picked (with a feather pick or a pipette) under a dissection microscope, and directly used for DNA extraction using an Ezup Column Animal Genomic DNA Purification Kit (Sangon Biotech, Shanghai, China) [45]. These genomes were prepared using an Illumina TruSeq DNA Sample Preparation Kit using manufacture’s protocols and sequenced with an Illumina NovaSeq 6000 platform, and were not subjected to mitochondrial probe hybridization [46] like those sourced from Gendron et al. 2023.

### Genome assembly

The USA generated raw sequencing reads were demultiplexed using the Illumina bcl2fastq conversion Software v1.8.4. and processed as described by Gendron et al., 2023. Briefly, sequencing reads were quality filtered using FastQC v0.11.5 [47] to remove adapters and low-quality bases. Contigs were assembled through de novo assembly using the SPAdes pipeline [48], Mitochondrial genomes were identified by BLASTing the full assembly against the NCBI nt database at ≥ 75% ID match, and then filtered those of non-mitochondrial origin. Assemblies were produced using SPAdes v3.13.0 using standard paired end inputs. Annotations were curated by hand by utilizing the BLAST algorithm to confirm gene identity. Reading frames were checked by translating each sequence and identifying start and stop codons by utilizing the Expasy web tool [49]. Successfully assembled genomes were determined by the presences of 12 PCGs, 22 tRNAs, 2 rRNAs, and the circularization of the genome. All successfully assembled genomes were oriented starting with the COX1 gene. Fragmented or failed assemblies were subjected to a pipeline where the raw reads were quality controlled by fastp [50] and aligned with a customized database containing 36 nematode mitogenomes using the NextGenMap [51]. The mapped reads were extracted by SAMtools [52] and MitoZ [53] was used for de novo assembly. For mitogenomes that were not circularized, we further employed seed-and-extend algorithms implemented in NOVOPlasty [54], using filtered raw reads as input. The resulted contigs were annotated in MITOS [55] and MitoZ [53], and results were manually curated. After the initial assemblies, these genomes were checked by hand, and hand-assembled into single contigs using Geneious [56]. All other genomes and their corresponding PCGs were sourced from the NCBI GenBank database or from previous work [22], with annotations based on either published work, or those provided by the GenBank database.

Together, 261 mitochondrial genomes (mitogenomes) were catalogued using a standardized and structured PCG and nematode classification system [22]. For all below described analyses, all genomes and genes were split into 2 main groups at the class level of 1) Enoplea (40 mitogenomes) and 2) Chromadorea (221). To facilitate a more detailed understanding of genome composition and structure and control for the overrepresentation of Chromadorea, genome analyses were further split into three subgroups at the infraorder level to include: 2A) Tylenchina (58 mitogenomes), 2B) Rhabditina (93), and 2C) Spirurina (68). Finally, the analyses were also performed for a subset of representative genomes (141 mitogenomes after removing redundant species to reduce the representation of oversampled taxa such as *Caenorhabditis* spp.) of the above groups pooled together to span 4) the entire nematode phylum. To facilitate below described analyses, each group of genomes was partitioned into datasets for the full genomes and for each of the 12 mitochondrial PCGs (COX1, COX2, COX3, CYTB, NAD1, NAD2, NAD3, NAD4, NAD4L, NAD5, NAD6, and ATP6).

### Phylogenetic analyses

Phylogenetic analyses were performed for each of the five above mentioned groups. Each of the 12 PCGs datasets for each of the five groups were aligned using the MAFFT aligner under settings –auto and – ‘adjustdirectionaccurately’ [57]. For phylogenetic comparison that involved all 12 shared PCGs, the individual gene alignments were concatenated to represent an ‘aligned protein coding mitochondrial genome (PCG-genome)’. This concatenated alignment alongside the 12 individual PCG alignments were then fed to PartitionFinderV.2 and the IQTREE pipelines [58, 59]. PartitionFinder was set to search for best fit evolutionary models by codon position for each individual protein coding gene alignment and the concatenated PCG-genome alignments. Returned partitions and model predictions were used in conjunction with the IQTree web tool [58] to create maximum likelihood trees for each gene in each category. Consensus trees using each of the 13 trees (12 individual PCG trees and the concatenated alignment PCG-genome tree) were used to determine the final phylogenetic structure of each respective group dataset (i.e., Nematoda phylum, Enoplea class, and Tylenchina, Rhabditina, and Spirurina suborders).

To address potential variation in nucleotide composition among linages and genes, each genome was analyzed for several characteristics including the complete base pair (bp) length of the mitochondrial genome, bp length of each PCG, %GC and %AT composition of the full genome and each PCG sequence. From these data, GC nucleotide skews (GC skew = (G—C)/(G + C)) were calculated for the full genomes (including all rRNA, tRNA, and non-coding regions), each PCG, and each codon position of each PCG.

To address potential variation in evolutionary rates among lineages and genes, each alignment (individual PCG and concatenated PCG alignments) and phylogenetic tree were examined with PAML for the prediction of pairwise mutation rates between each sequence (runmode = -2). This calculates predicted rates for both synonymous (S) and non-synonymous (N) mutations. Synonymous (dS) and non-synonymous (dN) substitution rates were determined using phylogenetic trees constructed under gene and codon specific substitution rates. PAML was run with a setting using a codon-based model under the codeml script, under the branch model (model = 2), and using the invertebrate mitochondrial codon usage database (icode = 4) as a reference [60]. Each sequence in a dataset had mutation rates calculated in reference to an outgroup to Nematoda consisting of two tardigrades (*Hypsibius dujardini* and *Ramazzottius varieornatus*) and three arthropods (*Limulus polyphemus*, *Anopheles gambiae*, and *Lithobius forficatus*) species to represent Tardigrada and Arthropoda respectively. Using these outgroups for our comparisons, increases the likelihood of mutational saturation results in the substitution analyses; however, it also provides a standardized comparison for the entire Nematoda phylum. The average of these five different mutation rates was used as a standard to describe relative mutation rates across the entire nematode phylum.

Due to the large phylogenetic distances covered in our analysis of substitution rates, we also included an analysis of mutational saturation for each PCG for the Enoplea, the Rhabditina, the Spirurina, and the Tylenchina clades. Mutational saturation was calculated for all resolved sites for all codon positions utilizing the DMABE7 software using invariant proportions based on individual gene alignments for each subset of sequences [61, 62].

### Statistical analyses

All statistical analyses were run in R v4.3. To begin investigating how nematode mitochondrial genomes might be shaped by different selection pressures, all taxa were assigned a set of nematode life traits (i.e., feeding habit, habitat, and reproductive strategies, SI Table 1) based on retrieved published taxonomic literature for the specific species further followed by a trophic scheme as described by Hodda (2022). For undescribed species, literature of close relatives within the same genus was used instead. Genome characteristics were tested against the life traits and taxonomic resolution using a Kruskal–Wallis test, blocked by each of our clades (Enoplea, Tylenchina, Spirurina, Rhabditina). Tests were also blocked at multiple taxonomic ranks (phylum, subclass, and suborder) to better capture the nuance of potential relationships and at what rank they were or were not significant. Due to multiple testing, all results were adjusted for false positive correction using FDR in R [63]. To examine the potential phylogenetic relationship for all the genome characteristics, Kruskal–Wallis tests were used to determine variance explained for, and significance of each characteristic (Genome Length, PCG Length, PCG proportion, %GC, and GC Skews) based on current taxonomic rankings. These tests were also performed for the substitution rates and codon position specific skews. To account for the uneven sampling of nematode genomes across the clades and life traits, the selection of the Kruskal–Wallis test was driven by its robustness to uneven sampling of tested groups and non-normal data distributions [64]. Post-hoc tests of the Kruskal–Wallis tests were performed using Dunn tests to identify where variation was most pronounced in each group of categorical variables [65].

## Results

### General patterns in mitogenome characteristics across the Nematoda phylum

#### Extent of sourced taxa

**Fig. 1:**
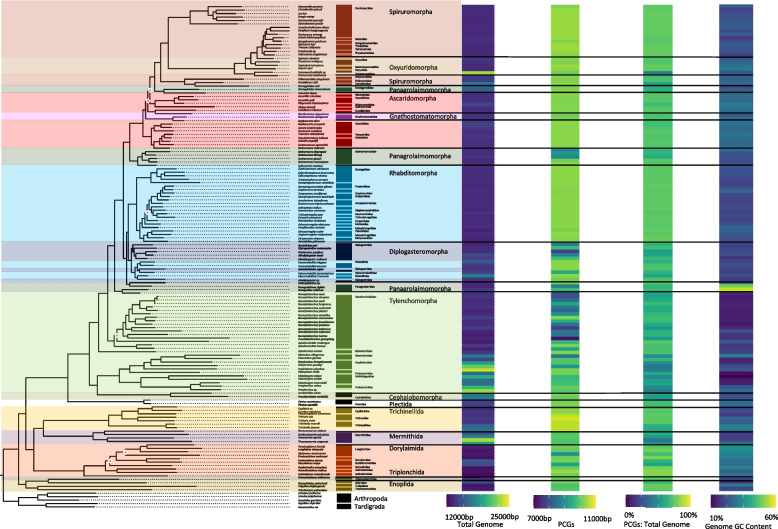
Phylum Mitogenome Characteristics. Heatmaps detailing each mitogenome characteristic (total genome length, PCG length, PCG %GC content, and total genome %GC content) are plotted alongside the tree ordered by the species order presented in the tree with a scale for each metric found at the bottom. The maximum-likelihood tree, of the Nematoda phylum, shown is based on 12 mitochondrial protein coding genes concatenated together to represent a subset of the analyzed nematode mitogenomes. Branch nodes have support values > 80 for all nodes except those denoted by a white and red diamond whose branch nodes fall below this support threshold. Branches and tips are colored by either order (for Enoplea clades and Plectida) or Infraorder for the Chromadoria clades. The tree is rooted with outgroups represented by members of Arthropoda and Tardigrada

We compiled and expanded a list of nematode mitochondrial genomes, representative of the Nematoda phylum, to include 261 complete genomes. This dataset encompasses various groups within the phylum: 7 from Mermithida, 16 Trichinellida, 1 Triplonchida, 13 Dorylaimida, 3 Enoplida, 2 Plectida, 58 Tylenchina, 93 Rhabditina, and 68 Spirurina. Although Rhabditida has been overrepresented, we were able to expand on genomes within the clades of Enoplia and Dorylaimia that are critical to understanding the root of the nematode tree [19, 66] as well as genomes within specific families (e.g., Aphelenchoididae, Steinernematidae, and Diplogastridae) to gain insights on genome characteristics at the level of deep and shallow branches. We demonstrated high support for Enoplida as the root of the phylum (Fig. 1). Furthermore, with 28 novel mitochondrial genomes produced for the suborder of Tylenchina, particularly for Panagrolaimomorpha, we recovered a non-monophyletic relationship among the Rhabditina and Spirurina suborders. Overall, the USA produced genomes had an average coverage of 63X coverage/million reads, and the Chinese produced genomes had an average coverage of 214X.

#### Genome size and composition

**Fig. 2:**
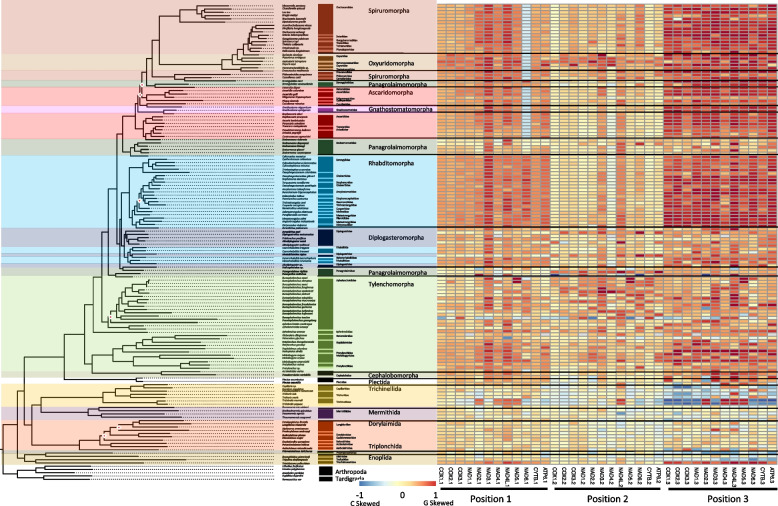
Phylum PCG Codon GC Skews. The heatmaps detail GC skews for each codon position, for each gene, in the concatenated genomes of 12 nematode mitogenome PCG sequences. Skews range from 1 to -1 representing high G content in the coding sequence the closer to 1 (red) and high C content the closer to -1 (blue). The maximum-likelihood tree, of the Nematoda phylum, shown is based on 12 mitochondrial protein coding genes concatenated together to represent a subset of the analyzed nematode mitogenomes. Branch nodes have support values > 80 for all nodes except those denoted by a white and red diamond whose branch nodes fall below this support threshold. Branches and tips are colored by either order (for Enoplea clades and Plectida) or Infraorder for the Chromadoria clades. The tree is rooted with outgroups represented by members of Arthropoda and Tardigrada

In agreement with the reported range of mitogenome sizes, the recovered, and sequenced mitogenomes for this study ranged from 12,438 – 26,195 bp (*Paractinolaimus indicus* and *Romanomermis culicivorax,* respectively) and all genomes were generally dominated by AT nucleotides (60 – 80%) resulting in GC content ranging from 20 – 40% (Fig. 1; SI Table 2) and this dominance was observed within all PCGs as well. Overall, the more basal lineages within Enoplia and Dorylaimia contained the genomes with the highest minimum and maximum combined PCGs length (min: 8,490 bp, max: 10,761 bp, avg: 10,208 bp) as well as the smallest and the largest total genome lengths, which were observed in the orders of Dorylaimida (*Paractinolaimus indicus*: 12,438 bp) and Mermithida (*Romanomermis culicivorax*: 26,195 bp), respectively. Enoplia and Dorylaimia also showed the lowest GC skews both for total genomes (avg: -0.064) and PCGs (avg: 0.040) (high C nucleotide content), and there was a general trend of increasing GC skews as we moved from basal branches to the shallower branches of Chromodoria (see Table 1 for infraorders) (avg: 0.34 and 0.38, respectively) (Fig. 2). With the smallest and longest total genomes, Enoplia and Dorylaimia showed the highest variation in total genome length (SI Table 2). In contrast, lineages within Chromadoria (Tylenchina, Rhabditina, and Spirurina), while displaying significant differences amongst themselves, they all shared highly similar sizes for both the total mitochondrial genomes (Avg. 14,557 bp) and combined PCGs (Avg. 10,049 bp), but, as a whole, Chromadoria genomes still did not significantly differ in size from non-chromadorian genomes (X^2^ = 0.73, p = 0.39) (SI Table 2,5,6). This result was confirmed by testing genome and PCG sizes at all rankings of taxonomic resolution to identify at which ranks they differentiated. While genome and PCGs sizes at the class and subclass levels were not statistically different (SI Table 5 & 6), differences became more evident and increased in explained variation with increasing taxonomic resolution, starting at the order level. Overall, neither feeding trait nor habitat played a significant role in the size of genomes and PCGs (SI Fig. 1, 2).

#### PCG coding strand compositional bias

**Fig. 3:**
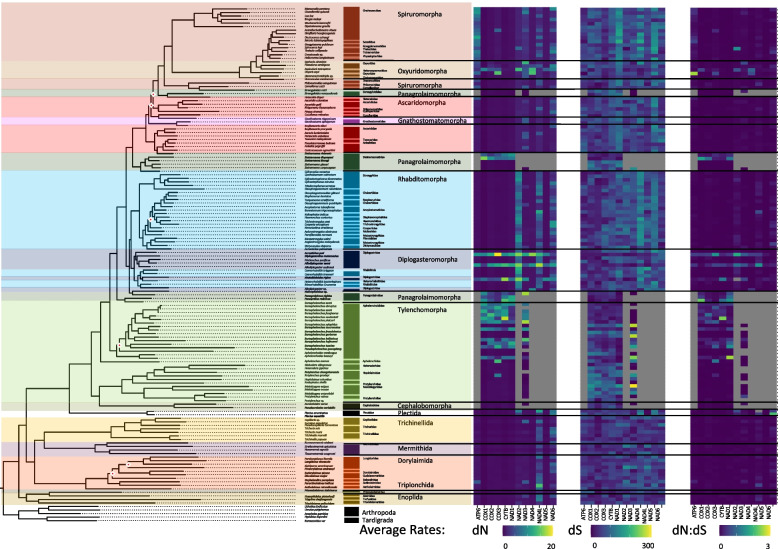
Phylum Mitogenome Substitution Rates. The heatmaps detail the non-synonymous (dN) and synonymous (dS) substation rates, and their ratio (dN:dS), calculated for each gene in the nematode mitochondrial genome based on a codon branch model using PAML. Rates were calculated relative to the outgroups Arthropoda and Tardigrada, with hotter colors denoting high substitution rates. Missing rates are due to a lack of enough resolved sites between the tested gene sequences. The maximum-likelihood tree, of the Nematoda phylum, shown is based on 12 mitochondrial protein coding genes concatenated together to represent a subset of the analyzed nematode mitogenomes. Branch nodes have support values > 80 for all nodes except those denoted by a white and red diamond whose branch nodes fall below this support threshold. Branches and tips are colored by either order (for Enoplea clades and Plectida) or Infraorder for the Chromadoria clades

To examine the potential role of phylogeny or life traits on the composition of the coding sequences, we measured GC skews (GC skew = (G—C)/(G + C)) within all PCGs at each codon position (1st, 2nd, and 3rd). Over the Nematoda phylum, coding strand composition varied from lightly C skewed (-0.178) for a handful of genomes to heavily G skewed (0.635) for the majority of mitochondrial genomes at the majority of codon positions (Fig. 2). A few exceptions occurred where codon positions were substantially C skewed but with little phylum level phylogenetic structuring. Feeding habit (e.g., bacterial feeder, fungal feeder, plant parasite), was highly significant in explaining PCG composition, largely driven by animal and plant parasitism, with the order of Trichinellida within Enoplea, known animal parasites, having shown consistent moderate to strong C skew, particularly in the 3rd codon position. *Trichinella* species (Trichinellidae) showed the strongest C skew (-0.870) (Fig. 3), but only for 7 out of the 12 PCGs (COX1, COX2, COX3, NAD3, NAD6, CYTB, and ATP6), followed by *Trichurus* species (Trichiuridae). Spirurina from the order of Rhabditida, another group of animal parasites, also showed evidence of C skews, but in contrast to Trichurida, every genome was C skewed in the 1st codon positions and only in the NAD6 PCG. Furthermore, the Ascaridomorpha, an infraorder of Spirurina, showed a consistent pattern of C skews at the 2nd codon position, but again not for all PCGs. Most of genomes within Rhabditina, many of which are animal parasites, were also C skewed at the 2nd codon position, but for specific to this suborder PCGs, particularly NAD4. Interestingly, Tylenchina, the third major suborder of Rhabditida, with many plant parasites, free-living, and insect-associated traits, had no codon-specific C skews, but overall plant parasites did generally show high G skews across the phylum. Overall, the strongest and most widespread C skews were observed in Enoplea, at the base of the tree in the 3rd codon position with a trend of increasing G skew in clades more distant from the root in the 1st and 3rd codon positions and a general trend of increasing C skews in the 2nd codon position particularly in taxa that were animal and plant parasitic (SI Fig. 1). Overall, these strong differences were reflected in the presence of significant differences in GC skews at every taxonomic level (SI Table 3), but relatively independent of the occupied habitat (SI Fig. 2).

#### PCG substitution rates

To investigate the possible influence of life traits on genome composition and coding strand biases and hence on the evolution of these mitogenomes, we calculated the synonymous and non-synonymous substitution rates for each PCG based on an average of pairwise comparisons to our 5 outgroup genomes. Non-synonymous substitution rates (dN) were generally an order of magnitude lower than synonymous substitution rates (dS) and ranged between 0.2 – 19.8 and 2.5 – 195.3, respectively (Fig. 3; SI Table 4 & 6). Missing results in the Tylenchina tree was due to the presence of internal stop codons within many of the coding sequences from these groups causing PAML to fail to find enough shared sites to compare. While the range of rates for both dN and dS substitutions was rather large, on average it was relatively similar across the Nematoda phylum. However, we did observe a significant relationship between dN rates and the expected life traits of species (SI Table 3 & 5), but most differences accounted for by inter-gene variation. The average dN rates across the phylum for each PCG showed relatively low standard deviations, with NADH genes being the most variable (e.g., NAD4L: 4.16 ± 2.42 versus COX1: 0.89 ± 1.56) (SI Table 3). Similar trends were observed for the dS with NAD1, NAD4, and NAD5 showing particularly high rates (NAD1: 77.03 ± 33.87, NAD4: 88.84 ± 33.64, NAD5: 85.76 ± 29.68). Among all PCGs, NAD4L had the highest dN rate (4.16) and COX1 the second lowest (0.89) rate. Interestingly, NAD5 had the lowest average dN rates, but had one of the highest average dS rates (85.756 ± 29.68). A comparison of the average dN:dS ratios across Nematoda unanimously had a ratio of < 1 indicating a low rate of evolution at the phylum level. We found that the average dN and dS rates were significantly explainable by both taxonomic rankings and life traits. For example, X^2^ values for dN rates were generally higher with increasing taxonomic resolution such as family and genus (Family: X^2^ = 142.92, p = 2.51e-09; Genus: X^2^ = 173.07, p = 0.006), as well as, with nematode feeding habits, habitats, and reproduction (Habit: X^2^ = 44.87, p = 9.75e-07, Habitat: X^2^ = 30.44, p = 0.023; Reproduction: X^2^ = 11.80, p = 0.003) (SI Fig. 3, SI Table 4 & 6). Microbivore piercers showed the highest dN rates across the phylum, but parasites, both animal and plant, showed higher dS rates. The highest dN rates by habitat were in the terrestrial and insect-associated species, which was more or less the same as the dS rates. Differences between reproduction strategies were more about differences in variation rather than means, but for the most part, amphimictic taxa possessed higher dN and dS rates.

### Enoplea class specific patterns in mitogenome characteristics

#### Extent of sourced taxa

**Fig. 4:**
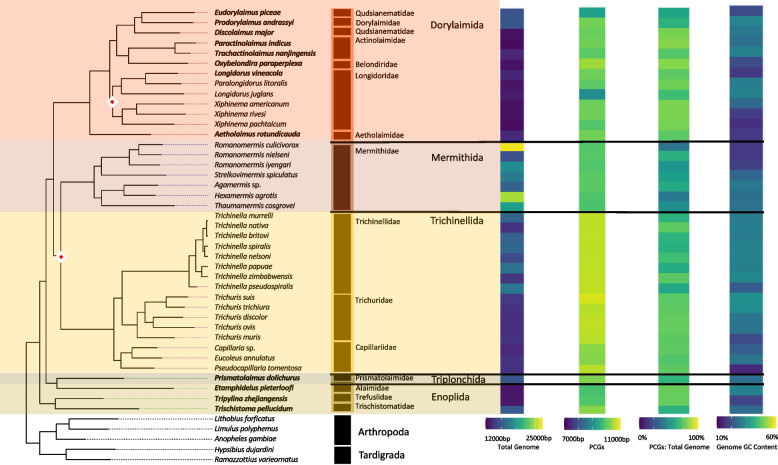
Enoplea Mitogenome Characteristics. Heatmaps detailing each mitogenome characteristic (total genome length, PCG length, PCG %GC content, and total genome %GC content) are plotted alongside the tree ordered by the species order presented in the tree with a scale for each metric found at the bottom. The maximum-likelihood tree, of the Enoplea class, shown is based on 12 mitochondrial protein coding genes concatenated together. Branch nodes have support values > 80 for all nodes except those denoted by a white and red diamond whose branch nodes fall below this support threshold. Branches and tips are colored by order. The tree is rooted with outgroups represented by members of Arthropoda and Tardigrada

The Enoplea (Enoplia and Dorylaima subclasses) spanned 40 mitochondrial genomes representing 5 orders: Enoplida (3 genomes), Triplonchida (1), Trichinellida (16), Mermithida (7), and Dorylaimida (13). The Trichinellida, while accounting for the greatest number of enoplid genomes, was one of the least diverse groups, with 13 of the 16 genomes representing only 2 genera in 2 families. In contrast, Dorylaimida was the most diverse, with its 13 genomes covering 10 genera in 6 families. The clade specific trees placed Enoplida and Triplonchida (the Enoplia subclass) as the root of the Enoplean tree (Fig. 4). In congruence with the phylum level tree, Dorylaimida was a sister clade (98 bootstrap likelihood) to Mermithida and Trichinellida, both of which were poorly resolved (78 bootstrap likelihood), in contrast to strong support of relationships within these two orders. Within Dorylaimida, the sequenced families split to two well-supported clades equivalent to suborders of Nygolamina and Dorylamina with the later indicated as paraphyletic.

#### Genome size and composition

Enoplid nematodes had both the smallest (*Paractinolaimus indicus,* 12,438 bp) and the largest (*Romanomermis culicivorax,* 26,195 bp) genomes of the entire dataset (SI Table 2). When examining just the PCGs, the clade contained the longest set of PCGs (10,761 bp) and accounted for the highest genome proportion (82%). Overall nucleotide composition across complete genomes vs. PCGs was similar (%GC of 28.71 ± 5.32 vs. 29.53 ± 5.66) but was the second most variable and the second lowest %GC (18.38%; Stdev = 5.32) of any of the Nematoda clades (Fig. 4). On average, while the complete genomes were C and A skewed and covered a wide range of GC and AT skews, the PCGs were G and T skewed and were less variable (SI Table 3). While significant variation was explained by the subclass level of resolution (SI Table 4), most of that variation came from differences between the Enoplia and Chromadoria subclasses, and we did not observe significant differences in genome size or composition between the two subclasses of Enoplea (Dorylaimia and Enoplia) (SI Table 2). We did observe a significant explanation of many genome characteristics, including genome size, genome %GC, and PCG size by life traits, particularly the feeding habit, with animal parasitic nematodes generally having larger genomes and PCGs and external plant parasites generally having higher %GC content (SI Table 5 & 6; SI Fig. 4). Habitat differences were largely driven by animal-associated taxa with the highest genome and PCGs sizes, and terrestrial taxa with higher %GC (SI Fig. 5). Genome size was the only characteristic to be significantly explained by reproduction (SI Table 5 & 6; SI Fig. 6).

#### Coding strand compositional bias

**Fig. 5:**
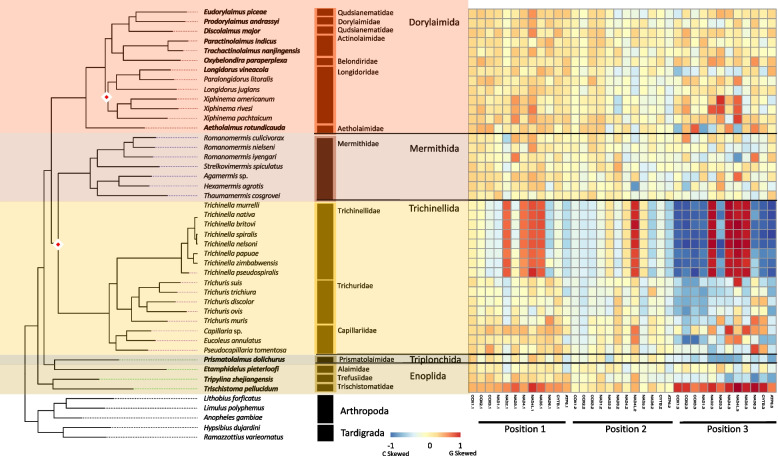
Enoplea PCG Codon GC Skews. The heatmaps detail the GC skews for each codon position, for each gene, in the concatenated genomes of 12 nematode mitogenome PCG sequences. Skews range from 1 to -1 representing high G content in the coding sequence the closer to 1 (red) and high C content the closer to -1 (blue). The maximum-likelihood tree, of the Enoplea class, shown is based on 12 mitochondrial protein coding genes concatenated together. Branch nodes have support values > 80 for all nodes except those denoted by a white and red diamond whose branch nodes fall below this support threshold. Branches and tips are colored by order. The tree is rooted with outgroups represented by members of Arthropoda and Tardigrada

Broadly, most enoplid taxa did not show a strong compositional bias across codon positions for any of the 12 PCGs (Fig. 5). However, there were many taxon-specific patterns. All species of *Trichinella* showed heavily skewed 3rd codon position with G skew within NAD2, NAD4, NAD4L, and NAD5 PCGs and C skew in the remaining PCGs. In addition, the 1st codon position G skew was observed for the NAD2, NAD4, NAD4L, and NAD5 PCGs. The C skewed 3rd position was generally conserved throughout all of the Trichinellida with the exceptions of *Trichuris muris* and *Capillaria* sp. The triplonchid *Prismatolaimus dolichurus* showed light C skew across all PCGs at the 3rd codon positions. While *Tripylina zhejiangensis*, a representative of Enoplida also showed some C skew in the 3rd codon position, the deepest branching *Trichistoma pellucidum* was heavily G skewed at the 3rd but also at the 1st codon positions. Dorylaimida and Mermithida were generally free of C skews, with some level of G skew observed for *Xiphinema* species but only within the NAD2, NAD3, and NAD5 genes at the 3rd codon position (Fig. 5). The GC skews were explained by both feeding habit and habitat life traits (SI Table 5 & 6), with animal parasites and animal-associated taxa generally showing the lowest PCGs GC skews, and microbivore processors and marine species the highest PCGs GC skews (SI Fig. 4, 5).

#### PCG substitution rates

**Fig. 6:**
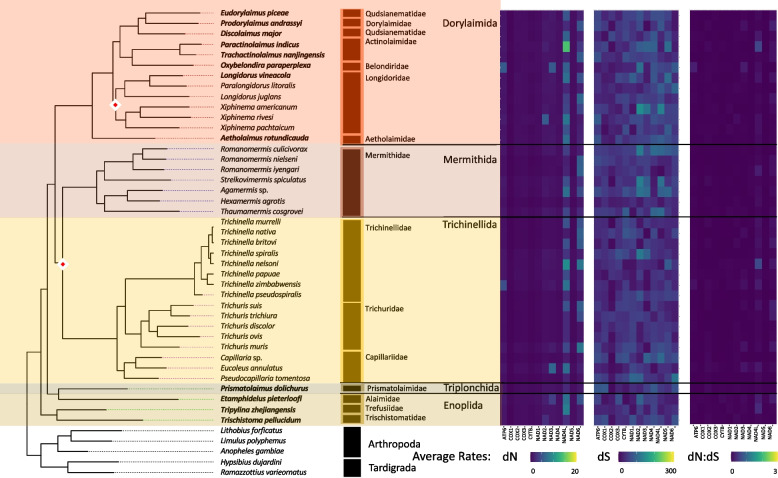
Enoplea Mitogenome Substitution Rates. The heatmaps detail the non-synonymous (dN) and synonymous (dS) substation rates, and their ratio (dN:dS), calculated for each gene in the nematode mitochondrial genome based on a codon branch model using PAML. Rates were calculated relative to the outgroups Arthropoda and Tardigrada, with hotter colors denoting high substitution rates. The maximum-likelihood tree, of the Enoplea class, shown is based on 12 mitochondrial protein coding genes concatenated together. Branch nodes have support values > 80 for all nodes except those denoted by a white and red diamond whose branch nodes fall below this support threshold. Branches and tips are colored by order

On average, enoplean mitochondrial PCGs showed some of the lowest dN rates (Fig. 3). The lowest rates were observed for the COX genes (Avg. COX1: 0.30, COX2: 0.58, COX3: 0.61) followed by the CYTB gene and NAD1 gene. The dN rates for NAD4L and NAD6 were generally higher across Enoplea (Fig. 6). The two taxa which showed the highest rates of evolution in Enoplea were found in the NAD4L genes of *Trichinella nelsoni* (Trichinellida) and *Paractinolaimus indicus* (Dorylaimida). The dS rates generally lacked strong patterns with perhaps lower rates in COX genes than other genes (Fig. 6). Substitution rates were not significantly different between the two subclasses of Enoplea, nor did they show significant relationships with life traits (SI Table 5 & 6; SI Fig. 4, 5). The Enoplea clade showed the greatest degree of mutational saturation of the clades with COX1 displaying the smallest degree of saturation (SI Table 7).

### Tylenchina suborder specific patterns in mitogenome characteristics

#### Extent of sourced taxa

The suborder Tylenchina was represented by 58 mitochondrial genomes across the infraorders of Tylenchomorpha (39), Panagrolaimomorpha (17), and Cephalobomorpha (2) covering a total of 9 nematode families. The majority of families were represented by Aphelenchoididae (23), Steinernematidae (11), and Meloidogynidae (8). Aphelenchoididae were dominated by *Bursaphelenchus* (17), with a few genomes from *Pseudaphelenchus* (2) and *Aphelenchoides* (3). Steinernematidae and Meloidogynidae consisted of only one genus each (*Steinernema* and *Meloidogyne,* respectively). Out of all 58 genomes of Tylenchina, 28 were sequenced for this study and included mostly Tylenchomorpha and Panagrolaimomorpha. In agreement with the phylum tree, Cephalobomorpha formed the base of the Tylenchina clade, but it wasn’t a well-supported branch. When examining only the Tylenchina genomes, the entire infraorder of Panagrolaimorpha was supported as a monophyletic sister clade to Tylenchomorpha. This was in contrast to the phylum level tree, where Panagrolaimidae was the only family of Panagrolaimomorpha to retain its relationship as a direct sister clade to Tylenchomorpha while the other families split into two clades (Steinernematidae and Strongyloididae) to become sister to Spirurina. Generally, the three Rhabditida suborders were more similar to each other in genome composition and nucleotide substitution rates than to Enoplea clades but did still show clade, as well as life trait specific, variation in genome characteristics and substitution rates. Overall, Tylenchina stood out for having the highest variation in genome characteristics and substitution rates out of the 3 suborder level clades. From the families within infraorders, the relationships between *Pseudaphelenchus* and *Bursaphelenchus* genera as well as among *Bursaphelenchus* species were unresolved. The only well-supported clade involved taxa belonging to the “*xylophilus*” group [67]. In addition, although Aphelenchoididae and Aphelenchidae families were recovered as sister clades, both were placed at the base of Panagrolaimidae. Most of the relationships among plant-parasitic taxa classified as the superfamily Tylenchoidea were well-supported. Pratylenchidae were observed as paraphyletic with *Rhadopholus* placed as sister to Hoplolaimidae and Heteroderidae. Within the infraorder of Panagrolaimomorpha, Steinernematidae formed two clades (*S. abbasi*/*S. carpocapsae* vs. other taxa).

#### General characteristics

**Fig. 7:**
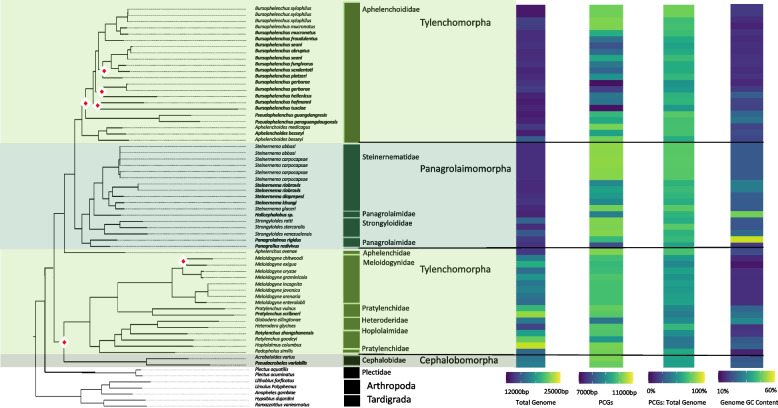
Tylenchina Mitogenome Characteristics. Heatmaps detailing each mitogenome characteristic (total genome length, PCG length, PCG %GC content, and total genome %GC content) are plotted alongside the tree ordered by the species order presented in the tree with a scale for each metric found at the bottom. The maximum-likelihood tree, of the Tylenchina suborder, shown is based on 12 mitochondrial protein coding genes concatenated together. Branch nodes have support values > 80 for all nodes except those denoted by a white and red diamond whose branch nodes fall below this support threshold. Branches and tips are colored by infraorder. The tree is rooted with outgroups represented by members of Arthropoda and Tardigrada

Broadly, there was little evidence of phylogenetic structuring of genome characteristics in Tylenchina. The mitochondrial genome size ranged from 12,946 – 25,228 bp accounting for both the 2nd smallest (*Bursaphelenchus xylophilus*) and 2nd largest (*Hoplolaimus columbus*) genomes of the Nematoda phylum. The PCGs lengths ranged from 7,155 – 10,330 bp and on average made up 62.34% of the mitochondrial genome. All *Bursaphelenchus* species, and a handful of *Steinernema* species, had some of the shortest cumulative length of PCGs (Fig. 7). The genomes had an average %GC of 21.57% ± 7.15 but showed the largest variation in nucleotide composition ranging from 13.01% to 56.06%. In line with the full genomes, PCGs were on average also G and T skewed and had a similar %GC content (21.49% ± 4.36). Although the genomes were generally G and T skewed, a large variation in both AT and GC skews across the suborder was observed (SI Table 2). However, in contrast to the full genomes, the range of GC and AT skews were heavily weighted towards G and A nucleotides (SI Table 2). Many of the characteristics showed significant relationship with life traits, both feeding habit and habitat, having some of the highest X^2^ values (SI Table 5 & 6; SI Fig. 7, 8). The high X^2^ values for %GC were driven by plant parasites possessing the lowest %GC and the largest genomes, while animal parasites had the highest GC skews. The %GC was also explained by habitat but was largely driven by differences in variation more than by averages, with terrestrial species characterized by lower %GC values. Moreover, parthenogenetic species had relatively larger genomes, although the PCGs lengths remained similar. In addition, parthenogenetic genomes were characterized by substantially lower %GC (SI Fig. 9).

#### Coding strand compositional bias

**Fig. 8:**
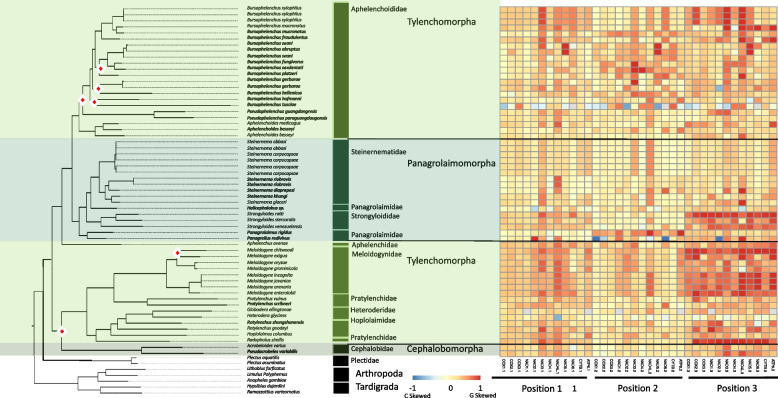
Tylenchina PCG Codon GC Skews. The heatmaps detail the GC skews for each codon position, for each gene, in the concatenated genomes of 12 nematode mitogenome PCG sequences. Skews range from 1 to -1 representing high G content in the coding sequence the closer to 1 (red) and high C content the closer to -1 (blue). The maximum-likelihood tree, of the Tylenchina suborder, shown is based on 12 mitochondrial protein coding genes concatenated together. Branch nodes have support values > 80 for all nodes except those denoted by a white and red diamond whose branch nodes fall below this support threshold. Branches and tips are colored by infraorder. The tree is rooted with outgroups represented by members of Arthropoda and Tardigrada

The coding strand compositional bias across codon positions for Tylenchina was less structured than any of the other clades, with high variation in GC skews observed at all three positions (Fig. 8). The strongest G skew was still observed in the 3rd codon position. The highest G skews across all PCGs were observed within the genera of *Strongyloides* (animal parasites) and *Meloidogyne* (plant parasites) representing Panagrolaimomorpha and Tylenchomorpha infraorders, respectively. Among tylenchomorphs, only one other species, *Radopholus similis* showed similar patterns, while the remaining species (e.g., *Pratylenchus)* had seemingly random light C skews at various genes. We also observed relatively high G skews in the 1st and 3rd codon positions of the plant parasitic *Bursaphelenchus xylophilus* and *B. mucronatus*. Tylenchomorpha also showed slight to moderate G skew at 1st codon positions for NAD3, NAD4, NAD4L, and NAD5, and at 2nd positions for NAD4L. Lastly, COX PCGs generally had the lowest G skews but only at the 1st and 2nd codon positions. Interestingly, C skew was generally absent, except for seemingly random genes as random codon positions for *Bursaphelenchus tusciae* and *Panagrellus redivivus.* PCGs GC skews explained by life traits had some of the highest X^2^ values as well. Within Tylenchina, PCG GC skews were driven by the significantly higher PCGs GC skews of parasites, both animal and plant. For habitat, terrestrial species showed higher skews than animal-associated skews. Lastly, skews were significantly explained by reproduction strategies with parthenogenic species showing higher skews than amphimictic species.

#### PCGs substitution rates

**Fig. 9:**
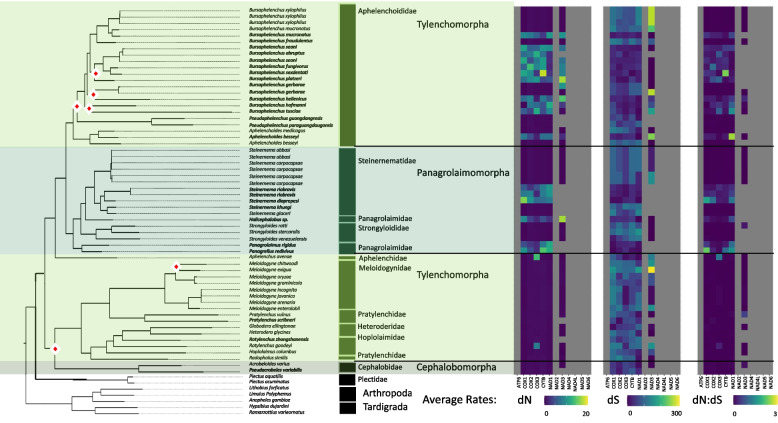
Tylenchina Mitogenome Substitution Rates. The heatmaps detail the non-synonymous (dN) and synonymous (dS) substation rates, and their ratio (dN:dS), calculated for each gene in the nematode mitochondrial genome based on a codon branch model using PAML. Rates were calculated relative to the outgroups Arthropoda and Tardigrada, with hotter colors denoting high substitution rates. Missing rates are due to a lack of enough resolved sites between the tested gene sequences. The maximum-likelihood tree, of the Tylenchina suborder, shown is based on 12 mitochondrial protein coding genes concatenated together. Branch nodes have support values > 80 for all nodes except those denoted by a white and red diamond whose branch nodes fall below this support threshold. Branches and tips are colored by infraorder

The mitochondrial genomes of Tylenchina did not show strong patterns of dN substitution rates across any of the genes (Fig. 9). However, relatively high dN rates (> 10) were observed in specific taxa (primarily *Bursaphelenchus* and *Steinernema*) for which the rates were high (10 -19) across all PCGs. The dS rates in Tylenchina were generally stochastically dispersed. The NAD3 gene showed a particularly wide range of variation from the highest (159.54) to the lowest (16.70) dS rates, but without any specific phylogenetically consistent pattern (Fig. 8). The dN:dS ratios most closely mirrored those of the dN rates (Fig. 9). The substitution rates for most of the NADH (especially NAD2, NAD4, NAD4L, NAD5, NAD6, and some of the NAD3) and ATP6 genes remained unresolved due to their high numbers of internal stop codons which PAML was unable to parse. Within Chromadoria, dN rates were significantly different for Tylenchina when compared to Spirurina and Rhabditina. While the average dN rates for Tylenchina were higher than the other two clades, the significant difference was largely due to the larger variation of dN rates present in Tylenchina than in the other two clades (SI Table 4 & 6). Within Tylenchina, dN rates were significantly explained by differences between parasites and microbivores (SI Table 5 & 6) with parasites characterized by lower dN rates and higher dS rates (SI Fig. 7). The Tylenchina clade showed the second highest degree of substitutional saturation with ATP6 showing the least degree of saturation of the mitochondrial PCGs (SI Table 7).

### Rhabditina subclass specific patterns in mitogenome characteristics

#### Extent of sourced taxa

The suborder of Rhabditina was represented by 93 mitochondrial genomes spanning two infraorders of Rhabditomorpha (84) and Diplogasteromorpha (9). The 17 total families were dominated by species of Chabertiidae (15), Rhabditidae (17), and Strongylidae (17). Diplogasteridae spanned 9 species in 5 genera. Genomes produced by this study were restricted to Diplogasteromorpha, which added 6 mitochondrial genomes to the previously available data. Rhabditina was the clade that was least dissimilar from the phylum tree, with Diplogasteromorpha remaining basal to Rhabditomorpha; however, we did observe that *Allodiplogaster* sp. and *Rhabditidoides rigina* changed in relation to the rest of the diplogastrid clade but remained basal to Rhabditomorpha.

#### General characteristics

**Fig. 10:**
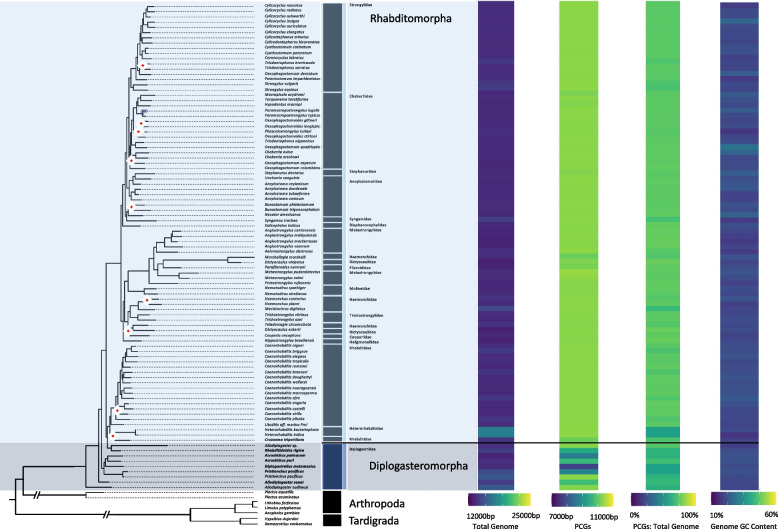
Rhabditina Mitogenome Characteristics. Heatmaps detailing each mitogenome characteristic (total genome length, PCG length, PCG %GC content, and total genome %GC content) are plotted alongside the tree ordered by the species order presented in the tree with a scale for each metric found at the bottom. The maximum-likelihood tree, of the Rhabditina suborder, shown is based on 12 mitochondrial protein coding genes concatenated together. Branch nodes have support values > 80 for all nodes except those denoted by a white and red diamond whose branch nodes fall below this support threshold. Branches and tips are colored by infraorder. The tree is rooted with outgroups represented by members of Arthropoda and Tardigrada

The size of mitochondrial genomes of Rhabditina ranged from 13,301 to 18,129 bp illustrating the narrowest range of genome sizes of all the examined clades with the smallest belonging to *Dictyocaulus eckerti* (13,311 bp) and the largest to *Heterorhabditis bacteriophora* and *Heterorhabditis indica* (18,129 bp) (Fig. 10). Total PCGs length ranged from 7,788 – 10,375 bp and accounted for an average of 73.25% of the mitochondrial genomes. This clade also had the lowest variation in nucleotide composition with an average %GC of 23.73% ± 1.7. The full genomes were on average G and T skewed (SI Table 2). PCGs, while of similar nucleotide composition, were exclusively G skewed. Many of the general genome characteristics of Rhabditina species were significantly lower compared to its sister clades (Spirurina and Tylenchina) for genome size, %GC content, and combined PCGs size; otherwise, characteristics generally fell between Spirurina and Tylenchina (SI Table 5 & 6). There were generally not differences associated with life traits.

#### Coding strand compositional bias

**Fig. 11:**
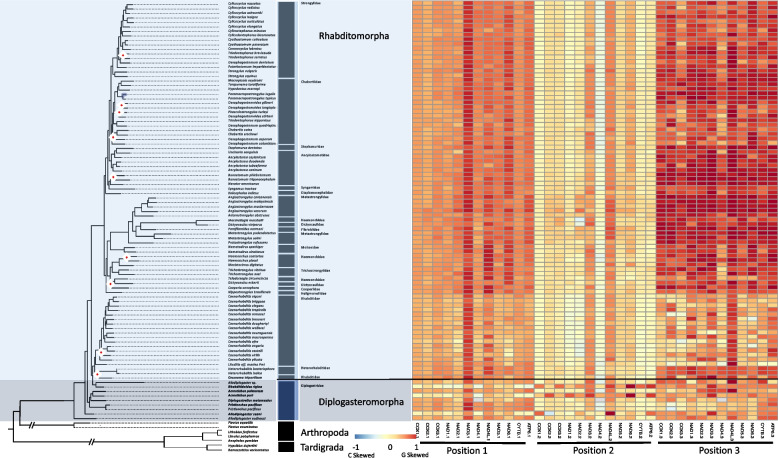
Rhabditina PCG Codon GC Skews. The heatmaps detail the GC skews for each codon position, for each gene, in the concatenated genomes of 12 nematode mitogenome PCG sequences. Skews range from 1 to -1 representing high G content in the coding sequence the closer to 1 (red) and high C content the closer to -1 (blue). The maximum-likelihood tree, of the Rhabditina suborder, shown is based on 12 mitochondrial protein coding genes concatenated together. Branch nodes have support values > 80 for all nodes except those denoted by a white and red diamond whose branch nodes fall below this support threshold. Branches and tips are colored by infraorder. The tree is rooted with outgroups represented by members of Arthropoda and Tardigrada

Broadly, Rhabditina strand bias was heavily G skewed (0.459 ± 0.281). However, when examining individual codon positions, we observed that the strongest G skews were concentrated in the 3rd codon position while the 2nd codon position was lightly C skewed (Fig. 11). The 1st codon position showed less compositional bias except for NAD3, which showed a strong G skew across the entire clade. NAD3 and NAD4L in the 2nd codon positions showed a consistent G skew across the entire clade. One of the strongest C skews in the clade was located in the 2nd codon position in the NAD3 gene. Lastly, Diplogasteromorpha observed C skews in the 1st codon position of *Acrostichus puri* and *Allodiplogaster seani* of almost all PCGs, in the 2nd codon positions except for NAD3 and NAD4L, and their 3rd codon position showed some of the lowest G skew of the suborder. PCG GC skews were significantly different from those of Tylenchina and Spirurina falling in-between the other two clades. PCG GC skews were also significantly explained by nematode feeding habit, with animal parasitic species showing much higher skews than their microbivore counterparts (SI Fig. 10), habitat, with animal associated species showing higher GC skews compared to terrestrial species (SI Fig. 11), and reproduction (SI Fig. 12), with amphimictic species showing higher GS skews than hermaphroditic.

#### PCG substitution rates

**Fig. 12:**
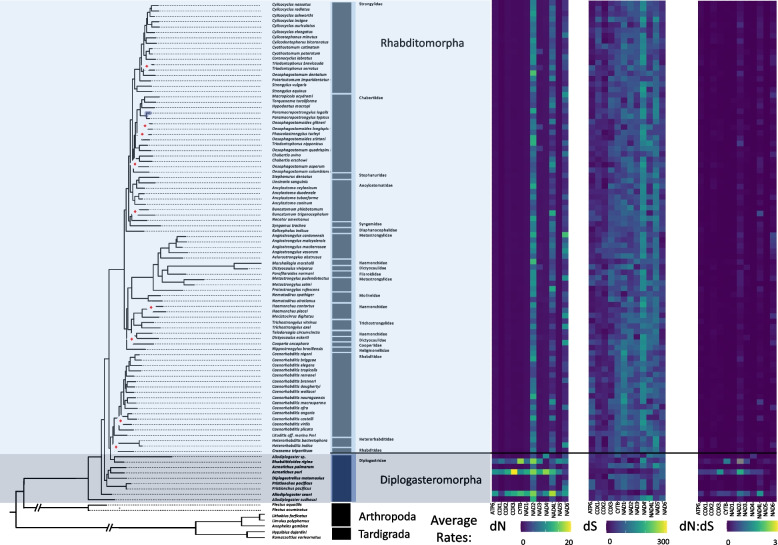
Rhabditina Mitogenome Substitution Rates. The heatmaps detail the non-synonymous (dN) and synonymous (dS) substation rates, and their ratio (dN:dS), calculated for each gene in the nematode mitochondrial genome based on a codon branch model using PAML. Rates were calculated relative to the outgroups Arthropoda and Tardigrada, with hotter colors denoting high substitution rates. The maximum-likelihood tree, of the Rhabditina suborder, shown is based on 12 mitochondrial protein coding genes concatenated together. Branch nodes have support values > 80 for all nodes except those denoted by a white and red diamond whose branch nodes fall below this support threshold. Branches and tips are colored by infraorder

The mitochondrial genomes of Rhabditina showed some taxon specific patterns of dN substitution rates (Fig. 12). Three members of Diplogasteromorpha (*Rhabditidoides rigina, Acrosticus puri, and Allodiplogaster seani)* had consistently high dN rates across all or most of the PCGs, with an average of 10 non-synonymous substitutions and with the highest dN rate seen in COX3 of *Acrostichus puri* (dN: 19.75; Fig. 12). Outside of these three taxa, the dN rates of Rhabditina did not show a phylogenetic structure. Instead, we observed dN rates largely determined by the PCG, with NAD2 (3.86), NAD4L (1.49), and NAD6 (1.62) showing consistently highest dN rates across the suborder. The dS rates were largely random with respect to phylogeny and traits. However, the rates varied among the PCGs with ATP6 (36.62 ± 25.5), COX1 (38.68 ± 16.5), COX2 (28.39 ± 16.1), and COX3 (55.42 ± 27.5) generally showing lower dS rates than the other genes across all taxa. The three taxa mentioned above also had relatively low dS (Fig. 12) and hence the highest dN:dS ratio. Rhabditina dN and dS rates did not show significant relationships with any life traits, making it stand out from its sister clades of Tylenchina and Spirurina. The lack of significance was largely due to low sample numbers in multiple habitat categories and the large variation in parasitic species and the small variation in the predator category. The Rhabditina clade demonstrated some of the least mutational saturation of the clades, but still identified substantial saturation in every gene. ATP6, COX1, and CYTB demonstrated the lowest degrees of saturation within the clade (SI Table 7).

### Spirurina subclass specific patterns in mitogenome characteristics

#### Extent of sourced taxa

The Spirurina suborder was represented by 68 full mitochondrial genomes representing the infraorders of Ascaridomorpha (34), Rhingonematomorpha (1), Gnathostomatomorpha (2), Spiruromorpha (25), Oxyuridomorpha (7), one genome from the family Dracunculidae (*Dracunculus medinensis*), and one from the family Philometridae (*Philmetroides sanguineus*). This was the only clade that did not benefit from any additional mitochondrial genomes from this study and entirely consisted of animal parasitic and amphimictic taxa that prevented an analysis of feeding habits, habitats, and reproductive strategies for this clade (SI Fig. 13, 14, 15). Spirurina experienced some of the largest differences between the topologies of trees generated for the entire phylum vs. the Spirurina clade. In contrast to the phylum tree indicating Ascaridomorpha as the most basal group of the clade, the Spirurina tree showed Oxyuridomorpha as the basal clade to the subclass. Furthermore, Gnathostomatomorpha was basal to Ascaridomorpha in the Spirurina clade tree, but in the phylum tree, Gnathostomatomorpha was paraphyletic with Ascaridomorpha.

#### General characteristics

**Fig. 13:**
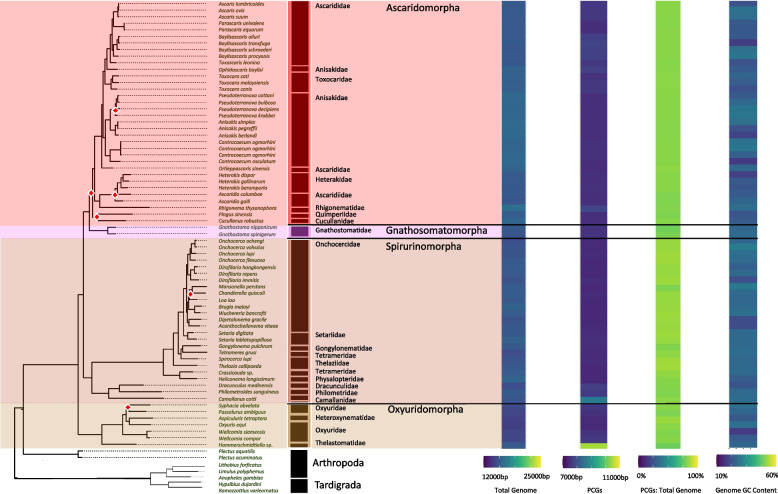
Spirurina Mitogenome Characteristics. Heatmaps detailing each mitogenome characteristic (total genome length, PCG length, PCG %GC content, and total genome %GC content) are plotted alongside the tree ordered by the species order presented in the tree with a scale for each metric found at the bottom. The maximum-likelihood tree, of the Spirurina suborder, shown is based on 12 mitochondrial protein coding genes concatenated together. Branch nodes have support values > 80 for all nodes except those denoted by a white and red diamond whose branch nodes fall below this support threshold. Branches and tips are colored by infraorder. The tree is rooted with outgroups represented by members of Arthropoda and Tardigrada

Genome size ranged between 13,591 – 24,563 bp with the smallest genome belonging to *Loa loa* and the largest to an undefined *Hammerschmidtiella* species (KY399989). However, the average genome size for Spirurina was the smallest out of all the clades investigated (Fig. 13). Total PCG lengths ranged from 10,105 – 10,431 bp and constituted an average of 72.95% of the mitochondrial genome. Along with similarly small sized genomes, Spirurina had relatively low variation in nucleotide composition although a relatively high average %GC content (27.56 ± 2.71) when compared to Tylenchina (21.57 ± 7.15) and Rhabditina (23.73 ± 1.72) clades (SI Table 2). Spirurina was most similar in nucleotide composition to the Enoplea clade (28.71 ± 5.32%GC).

#### Coding strand compositional bias

**Fig. 14:**
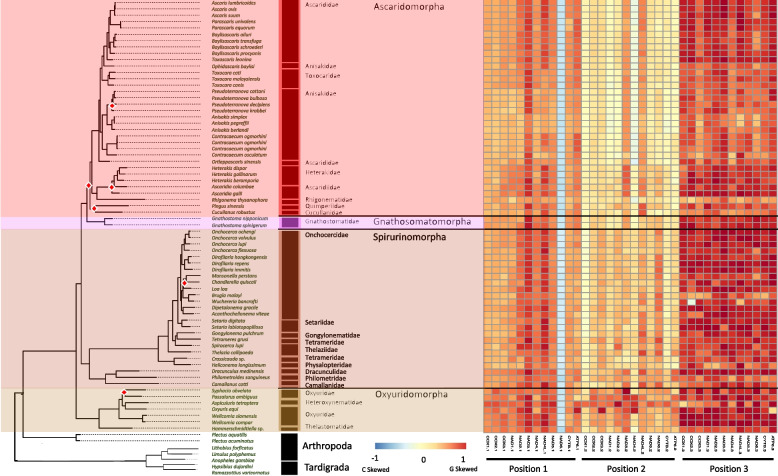
Spirurina PCG Codon GC Skews. The heatmaps detail the GC skews for each codon position, for each gene, in the concatenated genomes of 12 nematode mitogenome PCG sequences. Skews range from 1 to -1 representing high G content in the coding sequence the closer to 1 (red) and high C content the closer to -1 (blue). The maximum-likelihood tree, of the Spirurina suborder, shown is based on 12 mitochondrial protein coding genes concatenated together. Branch nodes have support values > 80 for all nodes except those denoted by a white and red diamond whose branch nodes fall below this support threshold. Branches and tips are colored by infraorder. The tree is rooted with outgroups represented by members of Arthropoda and Tardigrada

The complete Spirurina genomes were heavily G (0.437) and T (-0.389) skewed which was also mirrored in the PCG sequences, with all genomes and PCGs showing strong G and T skews. In addition, the clade showed the lowest variation in nucleotide skews (0.437 ± 0.072 GC skews and 0.389 ± 0.07 AT skews). Overall, there was considerable genome skew stability across the entire suborder with some infraorder level structuring. There was a general pattern of consistent GC skews across the entire suborder, with the 3rd codon positions most heavily G skewed for the great majority of taxa and all PCGs (Fig. 14). At the 1st codon positions, most genomes also showed a moderate to heavy G skew, but mostly for the NADH genes, especially, NAD3 and NAD4L. NAD6 was the only gene with a heavy C skew across all of the examined taxa. There was quite a bit of variation at the 2nd codon positions among the infraorders, with Oxyuridomorpha showing some of the highest G skew, Spiruromorpha very little skew, except for light to moderate G skew in NAD4L and NAD6, and Ascaridomorpha with some of the lightest G skew, except for the NAD3 and NAD4L genes, and the NAD4 gene with a C skew rather than a G skew. Spirurina had significantly higher PCG GC skews than Rhabditina or Tylenchina (SI Table 5 & 6). PCG GC skews also saw a significant relationship with the habitat life traits similar to the total genome GC skew, with much of the difference driven by the higher skews associated with the terrestrial mammal associated species.

#### PCG substitution rates

**Fig. 15:**
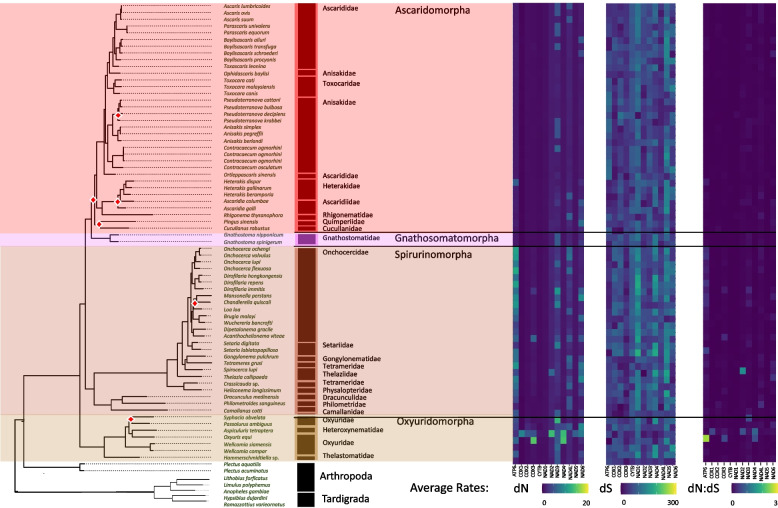
Spirurina Mitogenome Substitution Rates. The heatmaps detail the non-synonymous (dN) and synonymous (dS) substation rates, and their ratio (dN:dS), calculated for each gene in the nematode mitochondrial genome based on a codon branch model using PAML. Rates were calculated relative to the outgroups Arthropoda and Tardigrada, with hotter colors denoting high substitution rates. The maximum-likelihood tree, of the Spirurina suborder, shown is based on 12 mitochondrial protein coding genes concatenated together. Branch nodes have support values > 80 for all nodes except those denoted by a white and red diamond whose branch nodes fall below this support threshold. Branches and tips are colored by infraorder

In contrast to other clades’ patterns of dN rates, Spirurina showed some phylogenetic structure. The ATP6 gene in particular had some of the highest dN rates of the suborder but these high rates were reserved almost entirely to the most basal clades of Oxyuridomorpha (e.g., Oxyuridae) and Spiruromorpha *(e.g., Onchocera*: 26.46 ± 40.3 and *Dirofilaria*: 29.29 ± 47.6*)* (Fig. 15). Although two other genes (NAD4L and NAD6) in Oxyuridomorpha and Spiruromorpha also had relatively high dN rates, the rates were similar across the rest of the suborder. Two taxa from Oxyuridomorpha (*Aspiculuris tetraptera* and *Oxyuris equi*) were observed with relatively high dN rates across most of their PCGs, except for CYTB and NAD1 genes, with an average non-synonymous substitution rate of 19.58 ± 28.36. The dS rates in Spirurina were largely unstructured by either gene or lineage (Fig. 15). The highest dS rates were observed in NAD1 (110.43 ± 34.2), NAD4 (86.23 ± 43.6), and NAD5 (106.59 ± 37.6), but were somewhat clustered in Spiruromorpha. The dN:dS ratios for Spirurina were the most uniform of the clades, showing low ratios for almost every gene and lineage (Fig. 15). However, higher dN:dS ratios were observed in ATP6 (2.57) and NAD2 (0.75) particularly in *Oxyuris equi* and NAD2 of *Spirocerca lupi* (1.47). Within Chromadoria, dN rates showed a significant relationship with suborder, with Spirurina showing the lowest average dN rates (1.80 ± 0.85). While dS rates were not significantly explained by suborder. The Spirurina clade contained a similarly low degree of mutational saturation as Rhabditina, relative to Enoplea and Tylenchina. In contrast to Rhabditina, the less saturated genes were made up of NADH genes instead of COX genes.

### Overall trends and summary of Nematode mitochondrial genomes

The composition and structure of nematode mitochondrial genomes were particularly well explained by nematode lifestyle traits. This was most evident when comparing characteristics of the total genome versus characteristics of the PCG sequences. Specifically, %GC and compositional skews showed weak relationships with life traits when examining the entire genome, but when focusing on the actual coding sequences these traits showed much stronger relationships. Furthermore, we found that these relationships could at least in part be explained by a contrast between free-living and animal-associated/parasitic nematodes. Animal-associated and parasitic taxa generally showed lower %GC content in PCGs than in free-living taxa but also displayed higher GC skews in coding strand compositional bias. While not as statistically strong as compositional bias and %GC content, even dN and dS rates showed some relationship with life traits, particularly feeding habit, where again parasitic nematodes generally showed lower dN and higher dS rates than other categories of nematodes. Lastly, all the clades demonstrated a strong degree of mutational saturation but also saw variation between PCGs and clades.

In summary, the characteristics of nematode mitogenomes (i.e., genome size, compositional skews, and substitution rates) we measured showed some phylum level wide patterns (i.e., increasingly stronger GC skews in the 3rd codon position from the least to the most recently derived clades), but the strength of these patterns were more evident at narrower taxonomic ranks and tended to be more taxon specific than dictated by wider taxonomic relationships. More importantly, genome characteristics were equally or better explained by life traits (i.e. habit, habitat, and reproductive strategy) than by phylogeny.

## Discussion

Through the global distribution and placement across the entire food web, facilitated by high diversity of life traits, nematodes are not only important indicators of ecological health and stability [68], but also model metazoans to study the processes (e.g., selection) and mechanisms (e.g., environmental pressures and genome response) that shape genome evolution. The discrepancies between phylogenies derived from nuclear and mitochondrial DNA highlight the differential selection, giving rise to different evolutionary histories, at play on these genomes. These differences can largely be attributed to a variety of potential mechanisms including differences in replication methods and demands (e.g., single origin of replication, cell energy demands, strand displacement replication) [69], exposure to oxidative stress (e.g., high mutation chance of exposed single stranded DNA [ssDNA]) [70, 71], and the capacity for DNA repair (e.g., limited repair mechanisms that are dependent on nuclear gene expression) [72–75]. These mechanisms generate variation in mitogenome size, induce inversions, encourage compositional skews, and drive substitution rates [76]. In addition, they produce heteroplasmy (variation among mtDNA genome copies) within individual organelles, cells, and individuals [77–79]. Thanks to the unique selection pressures imposed upon mitochondrial genomes, they offer us a unique lens in which to study the potential role of both environmental and molecular mechanisms of mitochondrial genome evolution.

To begin to quantify the influence of selection pressures on the nematode mitochondrial genome, we collated 261 mitogenomes of nematodes from across the entire phylum. We analyzed these in the context of potential evolutionary pressures, exemplified by life traits adopted for survival. We hypothesized that the life traits would be among the most important factors explaining the variation of mitogenome characteristics. We expected that the broad spectrum of niches inhabited by nematodes, as well as previous evidence of repeated independent origins of traits (e.g., plant and animal parasitism) would facilitate mechanisms of rapid evolution, leading to potential adaptation, [14] and support observations of the punctuated nature of nematode evolution, even among congeneric species [80]. By testing metrics of evolutionary change (i.e., GC skews, genome structure, and substitution rates), we found that despite obfuscating factors, such as substantial mutational saturation, we still identified strong correlations between our metrics and life traits at different levels of phylogenetic relationships ranging from shallow (only specific narrow clades) to deeper (inclusive of wider taxonomic groups). These results help to support the potential influence of multi-level selection driven by interactions across organizational scales (from cells, to individuals, to populations, to species, to more inclusive clades). For example, replication pressure at the cellular level could drive the size of genomes to be small and hence replicate fast, but at the population level, small genomes with deleterious traits could be outcompeted by longer, more stable genomes [77]. Altogether, the findings in this manuscript work to provide a solid foundation of knowledge of the phylum wide, and clade specific, characteristics of nematode mitochondrial genomes.

### Consistency and deviation of Nematode mitogenome characteristics

The first suite of metrics we examined illustrated the general structure and composition (e.g., genome size, nucleotide content, and ratio of coding:non-coding DNA) of the nematode mitochondrial genomes. Mitochondrial genomes are under heavy pressure to maintain a constant supply of genetic material (transcription and mitosis ready) and undergo continuous turnover [74, 81]. Relative to nuclear genomes, in order to reduce replication time and energy cost, mitochondrial genomes are also expected to remain under selective pressures to have small, streamlined genomes that meet rapid energy production requirements in the cell. Taken together, a strong drive for consistency in genome characteristics (e.g., length of PCGs) is expected [82, 83]. However, this mitochondrial genome streamlining can also lead to gene loss or gene transfer to the nuclear genome [84] resulting in less consistent genomes across clades. In addition, multiple mtDNA copies in the mitochondria facilitate the presence of heteroplasmy among organelles, cells, and individuals [85, 86], albeit maintaining stability across generations [81]. Most of the previously reported variation in total mitochondrial genome size has been associated with non-coding regions, agreeing with our findings [87–91]. Compared to the variation observed for the whole genome lengths (Stdev.: 770 – 3141 bp), the variation in PCGs lengths was much narrower (Stdev: 387 – 811 bp) confirming that genome length variation in nematodes has been confined to the non-coding regions. Some species deviated from this pattern, but these were not associated with wide phylogenetic ranks (i.e., class and subclass) and instead with narrower ranks (e.g., family and genus) and thus were highly taxon specific (e.g., *Bursaphelenchus* spp. had lower than average PCG lengths). These narrower ranks were closely linked to life traits (especially feeding habits) that often explained the variation in characteristics better than phylogeny. In other words, environmental niche pressures likely played a major role, at least on par with phylogeny, in driving nematode mitogenome size.

Another structural metric indicative of the pressures shaping nematode mitogenomes is their high AT/low GC content. This content is expected as a result of the unidirectional nature of replication, mutations (often deaminations), and limited DNA repair. Together, these mechanisms generate heavy and light DNA strands, often each with its own compositional skew [34, 43, 69, 70, 75]. Instead of mirroring the phylogenetic relationships for genome size and relative coding content (strongly explained by narrow taxonomic ranks and life traits), we observed significant variation in %GC content explained by both wide (i.e., class, subclass, and order) and narrow (i.e., superfamily, family, and genus) taxonomic ranks. In addition, significant relationships with life traits were also observed. In short, contrary to genome size, nucleotide content was more evenly driven by both environmental niche pressures and phylogenetic relationships.

The variation in these metrics’ relationships with different taxonomic ranks (wide vs. narrow) suggests the presence of multi-level selection pressures operating on nematode mitogenomes at both deep and shallow phylogenetic scales. Furthermore, the support for multi-level selection becomes even more evident when examining the variation in the significance of life traits across the phylum. For example, the significance of feeding habit and habitat in explaining genome and PCG %GC was largely driven by two wide clades: Enoplea and Tylenchina. Likewise, although reproduction type did not explain genome size across the entire phylum, it was significant when tested within clades (i.e., in Enoplea, Tylenchina, and Rhabditina). While %GC content was significantly affected by wider phylogenetic scales, the variation in the majority of our metrics of general genomic structure and composition were best explained by congeneric species at shallow scales and by life traits. These patterns may be a result of strong environmental niche pressures that specific nematode taxa must adapt to. For example, it has been documented that genome size in animal parasitic taxa can be directly selected by environmental factors such as host body temperature [92]. This suggests that niche pressures likely drive evolutionary change of specific genomes rather than being driven by factors that apply across wide phylogenetic scales.

In summary, the general characteristics of the nematode mitochondrial genomes show great consistency with observations made of other nematode and metazoan mitogenomes. On the other hand, our data also provides further evidence to the literature that the nematode mitogenome displays remarkable plasticity [18, 27, 34, 55, 93], encouraging the generation of genetic diversity. Furthermore, we observed that these characteristics were selected for/against at multiple phylogenetic scales, both narrow and wide, largely governed by species specific niche selection pressures. However, while these metrics of general mitogenome structure and composition provided insights into the potential evolutionary pressures driving nematode mitochondrial genomes, they represented metrics that were too broad to effectively discriminate between the different mechanisms (e.g., genome size could be due to streamlining vs. expansion) [77, 94, 95] by which selection pressures shaped the observed characteristics.

### Drivers of coding strand compositional bias

The next metric we chose to evaluate the evolutionary pressures on nematode mitogenomes were strand specific compositional skews, specifically coding strand skews. Strand specific compositional skews can result from high replication rates accompanied by spontaneous deaminations and other mistakes in editing during both replication and transcription ultimately favoring the accumulation of T and G in the coding strand [95–99]. Generally, nematode PCGs possessed high GC skews, in agreement with previous literature identifying Nematoda as unique among metazoans for their strong skews [34]. The high skews have been hypothesized to be a result of the rapid generation cycles in nematodes compared to other metazoans [100], and generally we observed lower skews in Enoplea who are known for their relatively longer generation cycles [101]. Indeed, high GC skews were significantly explained by all taxonomic ranks and by both feeding habit and habitat when examining the entire phylum. This result provided support, similar to the general genome characteristics, that strand compositional skews may be the result of the multi-level selection operating on different clades across the whole phylum. Furthermore, when examined at narrower phylogenetic scales, we observed that although the effect of life traits on whole mitogenomes was somewhat variable, the effect on PCGs was highly significant for all clades supporting the idea that GC skew is likely the byproduct of gene expression [96].

The compositional biases we observed in the coding strand of PCGs and their link to the multi-level selection of gene expression was further emphasized when we examined codon position specific skews. Generally, the overall high PCG compositional skews were largely driven by high 3rd codon position skews, in line with previous observations of compositional bias with strongly G skewed codon positions seen in invertebrates [95, 102–104], and specifically linked to mutation potential in nematodes [29]. This position specific skewing has been hypothesized to result from evolutionary selection pressures on codon usage, ultimately influencing gene expression, with the 3rd position generally showing the most variability. This is due to the fact that the 3rd position tends to undergo higher rates of mutation. This feature of gene expression is compensated/facilitated by the fact that the majority of 3rd positions act as fourfold degenerate sites for amino acid translation [29, 105], and may be a major driver of genome evolvability [106].

Interestingly, we also observed relatively strong GC skewing in the 1st codon position for the Spirurina and Rhabditina clades. The GC content of the coding sequences of nematode nuclear genomes has been shown to correlate with different codon usage frequencies, with a shift from amino acids encoded by WWN (AA, AT, TA, or TT in the 1st and 2nd nucleotide positions) codons to SSN (GG, GC, CG, and CC) codons driven by changes in the 1st and 2nd codon positions [107]. If there are similar significant pressures on codon usage in mitochondrial genomes, this might explain the presence of skews in the 1st codon position in addition to the four-fold degenerate 3rd positions. The lack of the 1st codon position specific skews in the more basal Enoplea (and to a lesser extent Tylenchina), in contrast to the more recently derived clades, demonstrates strong codon usage selection variation among the clades. This highlights the variability of selection on different nematode mitochondrial genomes and hence supporting the idea of multi-level selection at play in the Nematoda phylum.

Heavy skews can also provide insight into the evolutionary history of a genome as they readily facilitate the identification of strand gene inversions [95]. In regard to some of the issues in using mitochondrial genomes to resolve the deeper branches of the nematode phylogenetic tree, we observed likely strand inversions of genes in many taxa. This can be a major factor in driving differences between mitochondria and nuclear derived phylogenies as the diploid nature of nuclear genomes significantly limits the inheritance of these kinds of events [108]. In agreement with previous work on Enoplea [109], we observed more potential inversions in Enoplia (i.e., Trichinellida) likely resulting from ORIs (origin of replication inversions). Furthermore, ORI events alter the strand exposed during expression and can be another factor influencing the accumulation of skews in coding sequences and often facilitating synonymous mutational saturation [99]. These inversions of strand asymmetry highlight the role of replication rather than transcription contributing to the evolution of mitochondrial genomes and helps to explain some of the confounding evolutionary relationships in Nematoda as inversions can obscure or even erase phylogenetic signals among sequences [99, 110].

Taken together, the observed patterns of strong coding strand compositional skews are likely, in part, a result of selection pressures placed on both replication and gene expression. The strong relationships with life traits, especially feeding habits (driven by differences between plant parasites and microbivores) and habitats (driven by differences between animal-associated and terrestrial taxa), emphasizes the multi-level selection by way of differing environmental niche pressures. However, nucleotide compositional skews are largely limited to capturing large scale trends, and generally are unable to identify subtler motifs and may not necessarily reflect all evolutionary selection pressures [99].

### Punctuated nature of Nematode substitution rates in mitochondrial coding sequences

The last metrics we chose to analyze were measures of synonymous (dS) and non-synonymous (dN) nucleotide substitution rates for each PCG in the mitogenome. Because mutations are among major mechanisms of evolutionary change, substitution rates can provide the most direct measure of the selection pressures shaping genomes [99]. While mutation induced substitutions are the mechanism, the selection pressures that drive the establishment or purification of a mutation in nematode populations remain unknown. Taxonomic ranks (both wide and narrow) together with life traits were strong predictors of substitution rates with the greatest differences in dS rates observed at the class level, and dN rates at the superfamily level. In terms of life traits, habitat best explained dS rates, while feeding habit best explained dN rates. Overall, nematode mitogenomes displayed strong purifying selection (< 1 dN:dS ratios when compared to their closest relatives of Arthropoda and Tardigrada), in line with general expectations for mitochondrial genomes [111–113] likely indicating strong environmental pressures limiting genetic diversity within most of the species.

The most widely accepted model of mitochondrial genome replication involves asymmetric strand displacement [114] (but see rolling-circle replication in *C. elegans*) [115] resulting in prolonged exposure of ssDNA to mutations via oxidizers present in the mitochondrial cytoplasm. Although some animals (e.g., mammals) have developed a system with both leading and lagging replication [98], mutations still occur [76], largely due to limited capacity for DNA repair [73, 75, 116]. These mechanisms often result in substitution/mutational saturation of the coding sequences, which we observed throughout the Nematoda mitochondrial PCGs, and can confound phylogenetic relationships [29, 99]. Particularly, we observed extremely high dS rates indicative of mutational saturation and partially resulting from the comparison to a standard non-Nematode outgroup. These high rates can likely be attributed to the large phylogenetic distance between nematode taxa and the chosen outgroup, and makes it difficult to determine the actual substitution rates. However, the susceptibility of mtDNA to mutations would provide another potential mechanism through which nematode mitogenomes can accumulate diversity. In fact, despite this degree of unreliability, we still observed significant relationships between taxonomy/life traits and both dS and dN rates. Furthermore, the strong purifying selection we observed demonstrates a process that is likely compensating for the accumulation of deleterious mutations to maintain functionality as has been observed in *C. briggsae* [117].

Alternatively, we also identified likely substitution hotspots in several lineages, with the most prominent observed in our long-standing (10–20 years) nematode cultures providing more direct evidence (high dN:dS ratio) for the capacity of rapid nematode evolution. A likely mechanism at play here could be through positive selection and/or selective sweeps. However, measures of these events are susceptible to false positives induced by changes to the effective population size through events such as bottlenecks and requires more in-depth investigation to resolve [118, 119]. While some of these high substitution rates can be attributed to the mutational saturation of these genomes alongside the comparison to the non-Nematode outgroup, we still observed significantly higher dN rates among these taxa than in any other groups. This rapid evolution may be driven by the differences in effective population sizes between environmentally sourced genomes and those sourced from cultures. While there has been some debate on the impact effective population sizes have on invertebrate mitochondrial genetic diversity, it has also been shown that the relative strength of purifying selection and effective population size are correlated in nematodes [112, 120], and can undergo rapid changes through events such as bottlenecking [115, 116, 121]. This effect may also result from a founder’s effect driven by mitochondrial heteroplasmy facilitating variation in mtDNA which could then become more prevalent in nematode populations experiencing repeated bottlenecks, such as the establishment of a new culture [77, 79, 112, 122]. Another potential mechanism is the architectural rearrangement of the genome through mechanisms such as ORI events, likely observed in Enoplea, that alter selection pressures on the coding sequence opening them up for mutational bursts [99]. These mechanisms would explain how, despite our evidence of strong purifying selection nematodes are still able to diversify and evolve in relatively short evolutionary time frames. Furthermore, in contrast to the high conservation of COX genes, we observed higher than average dN:dS ratios (though still not > 1) in NAD2, NAD4L, NAD6, and ATP6 similar to other findings when comparing parasitic nematode genera [123]. Taken together, nematode mitochondrial genomes demonstrate the flexibility to accumulate and purge mutations rapidly. Again, this flexibility is likely driven by multi-level selection pressures (e.g., habitats and feeding habits), utilizing molecular mechanisms to generate diversity at all levels of biological organization (e.g., species and genes) and then select (via purification) to avoid mutational meltdowns [124].

In conclusion, it is well known that mitochondrial genomes of most eukaryotes deviate from neutral selection models with strong patterns of directional selection that is highly related to the fitness of individuals [77, 125]. The phylum Nematoda provides us with supporting evidence of strong multi-level selection involving both environmental niche and phylogenetic relationships in the shaping of nematode mitochondrial genomes. The significance of genome metrics (e.g., genome size, GC skews, substitution rates) representing evolutionary selection pressures, life traits (i.e., habit, feeding habitat, and reproduction), and phylogenetic distance at small scales (e.g., genus rank) highlights the dual role niche pressures and population dynamics can have in driving the evolutionary rate and direction of species.

## Conclusions

The strong relationship with life traits may be a function of both adaptability and evolvability of nematode mitochondrial genomes. These traits are likely the product of the combination of variability and flexibility of nematode mitochondrial genomes [27, 35, 105]. This linkage provides a rationale for the unique characteristics of Nematoda compared to other metazoans. For example, this relationship was particularly evident for parasitic nematodes as opposed to free-living, with parasitic life styles highly associated with strong and rapid evolution. In fact, these mechanisms could provide a potential explanation for why parasitism shows multiple independent origins across Nematoda [14]. Similar to other invertebrates [126], this capacity for rapid evolution would also explain the punctuated nature of various nematode lineages and the long evolutionary histories observed among congeneric taxa [80, 113]. Overall, we observed genomes capable of rapid evolution, leading to potential adaptation (e.g., punctuated positive selection, multiple independent parasitism evolution, and high plasticity in genome features), but also selected for conservation and consistency to meet energy demands (e.g., consistency of the presence of GC skews in non-enoplean clades, stability of PCG sizes, and presence of strong purifying selection).

This study provides the first systematic insights of what we believe to be the most likely mechanisms influencing the evolution of the nematode mitochondrial genomes. However, many of the mechanisms we propose in this study still require significantly more investigation that can better consider the factors such as mutational saturation which can obfuscate these evolutionary relationships. While the mechanisms driving nematode mitochondrial evolution are not fully clear, their interaction with the diverse and abundant lifestyles of nematodes, likely result in the observed unique genomic compositions and structures. The heavy influence of life traits and environmental selective pressures on the nematode mitochondrial genomes continues to provide an opportunity for more extensive comparative evolutionary genomics, where with high diversity and capacity for rapid evolution, nematodes can help to shed light on how exactly species evolve and adapt to their environments potentially offering solutions to ecological disruptions driven by climate change or nematode pest management.

### Supplementary Information

Additional file 1: Table S1. Metadata Summary Table. Summary of nematode species mitochondrial genomes used for the study and their accompanying metadata.

Additional file 2: Table S2. Mitogenome Characteristics and Clade Specific Statistical Tests. Summary of ranges and stats for each analyzed genome characteristic organized by specific clades. Characteristics are accompanied by clade specific Kruskal-Wallis tests of mitogenome characteristics.

Additional file 3: Table S3. Genome Substitution Rates and Clade Specific Statistical Tests. Summary of ranges and stats for dN and dS substitution rates for each gene present in the nematode mitogenome. Rates are organized by specific clades. Substitution rates are accompanied by clade specific Kruskal-Wallis tests of mitogenome substitution rates.

Additional file 4: Table S4. Phylum Taxonomic and Lifestyle Statistical Tests. Phylum level summary of Kruskal-Wallis tests for taxonomic rank or life traits in predicting mitogenome characteristics and substitution rates.

Additional file 5: Table S5. Clade Specific Lifestyle Statistical Tests. Summary of clade specific Kruskal-Wallis tests for life traits in predicting mitogenome characteristics and substitution rates.

Additional file 6: Table S6. Summary of Statistical Results for Each Clade. A summary of results of significance for each clade specific Kruskal-Wallis test of genome characteristics and substitution rates set by each life trait of feeding habit, habitat preference, and reproductive strategy.

Additional file 7: Table S7. Mutational Saturation Statistical Tests. Summary of clade specific tests of mutational saturation for each of the 12 PCGs found in Nematode mitochondrial genomes. Mutational saturation was tested by clade for each PCG (Enoplea, Rhabditina, Tylenchina, and Spirurina).

Additional file 8: Fig. S1: Nematode Mitogenome Characteristics by Feeding Habit. Box and whisker plots for total genome and PCG characteristics for A) size, B) %GC content, C) GC compositional skew, and D) substitution rates for PCG sequences for the Nematoda phylum. Medians and quantiles were calculated for each characteristic based on the life trait classification for feeding Habit. Phylum level feeding habit was significant for all characteristics.

Additional file 9: Fig. S2: Nematode Mitogenome Characteristics by Habitat. Box and whisker plots for total genome and PCG characteristics for A) size, B) %GC content, C) GC compositional skew, and D) substitution rates for PCG sequences for the Nematoda phylum. Medians and quantiles were calculated for each characteristic based on the life trait classification for preferred Habitat. Phylum level habitat was significant for all characteristics expect dN rates.

Additional file 10: Fig. S3: Nematode Mitogenome Characteristics by Reproduction. Box and whisker plots for total genome and PCG characteristics for A) size, B) %GC content, C) GC compositional skew, and D) substitution rates for PCG sequences for the Nematoda phylum. Medians and quantiles were calculated for each characteristic based on the life trait classification for Reproduction strategy. Phylum level reproduction strategy was not significant for any characteristic.

Additional file 11: Fig. S4: Enoplea Mitogenome Characteristics by Feeding Habit. Box and whisker plots for total genome and PCG characteristics for A) size, B) %GC content, C) GC compositional skew, and D) substitution rates for PCG sequences for the Enoplea class. Medians and quantiles were calculated for each characteristic based on the life trait classification for feeding Habit. Enoplea feed habit was only significant for genome size.

Additional file 12: Fig. S5: Enoplea Mitogenome Characteristics by Habitat. Box and whisker plots for total genome and PCG characteristics for A) size, B) %GC content, C) GC compositional skew, and D) substitution rates for PCG sequences for the Enoplea class. Medians and quantiles were calculated for each characteristic based on the life trait classification for preferred Habitat. Enoplea habitat was significant for all characteristics expect for dN and dS rates.

Additional file 13: Fig. S6: Enoplea Mitogenome Characteristics by Reproduction. Box and whisker plots for total genome and PCG characteristics for A) size, B) %GC content, C) GC compositional skew, and D) substitution rates for PCG sequences for the Enoplea class. Medians and quantiles were calculated for each characteristic based on the life trait classification for Reproduction strategy. Enoplea reproduction strategy was only significant for genome size.

Additional file 14: Fig. S7: Tylenchina Mitogenome Characteristics by Feeding Habit. Box and whisker plots for total genome and PCG characteristics for A) size, B) %GC content, C) GC compositional skew, and D) substitution rates for PCG sequences for the Tylenchina suborder. Medians and quantiles were calculated for each characteristic based on the life trait classification for feeding Habit. Tylenchina feeding habit was significant for genome %GC, Genome GC skews, and PCG GC skews.

Additional file 15: Fig. S8: Tylenchina Mitogenome Characteristics by Habitat. Box and whisker plots for total genome and PCG characteristics for A) size, B) %GC content, C) GC compositional skew, and D) substitution rates for PCG sequences for the Tylenchina suborder. Medians and quantiles were calculated for each characteristic based on the life trait classification for preferred Habitat. Tylenchina habitat was significant for genome %GC, total PCG size, PCG proportion of the genome, PCG GC Skews, and dN rates.

Additional file 16: Fig. S9: Tylenchina Mitogenome Characteristics by Reproduction. Box and whisker plots for total genome and PCG characteristics for A) size, B) %GC content, C) GC compositional skew, and D) substitution rates for PCG sequences for the Tylenchina suborder. Medians and quantiles were calculated for each characteristic based on the life trait classification for Reproduction strategy. Tylenchina reproduction strategy was significant for genome size, genome %GC, PCG proportion of the genome, Genome GC skews, and PCG GC skews.

Additional file 17: Fig. S10: Rhabditina Mitogenome Characteristics by Feeding Habit. Box and whisker plots for total genome and PCG characteristics for A) size, B) %GC content, C) GC compositional skew, and D) substitution rates for PCG sequences for the Rhabditina suborder. Medians and quantiles were calculated for each characteristic based on the life trait classification for feeding Habit. Rhabditina feeding habits were significant for PCG proportion of the genome, genome GC skews, and PCG GC skews.

Additional file 18: Fig. S11: Rhabditina Mitogenome Characteristics by Habitat. Box and whisker plots for total genome and PCG characteristics for A) size, B) %GC content, C) GC compositional skew, and D) substitution rates for PCG sequences for the Rhabditina suborder. Medians and quantiles were calculated for each characteristic based on the life trait classification for preferred Habitat. Rhabditina habitats were significant for PCG proportion of the genome, genome GC skews, and PCG GC skews.

Additional file 19: Fig. S12: Rhabditina Mitogenome Characteristics by Reproduction. Box and whisker plots for total genome and PCG characteristics for A) size, B) %GC content, C) GC compositional skew, and D) substitution rates for PCG sequences for the Rhabditina suborder. Medians and quantiles were calculated for each characteristic based on the life traits classification for Reproduction strategy. Rhabditina reproductive strategies were significant for genome size, PCG proportion of the genome, genome GC skews, and PCG GC skews.

Additional file 20: Fig. S13: Spirurina Mitogenome Characteristics by Feeding Habit. Box and whisker plots for total genome and PCG characteristics for A) size, B) %GC content, C) GC compositional skew, and D) substitution rates for PCG sequences for the Spirurina suborder. Medians and quantiles were calculated for each characteristic based on the life traits classification for feeding Habit. Spirurina feeding habits were not significant for any characteristics.

Additional file 21: Fig. S14: Spirurina Mitogenome Characteristics by Habitat. Box and whisker plots for total genome and PCG characteristics for A) size, B) %GC content, C) GC compositional skew, and D) substitution rates for PCG sequences for the Spirurina suborder. Medians and quantiles were calculated for each characteristic based on the life traits classification for preferred Habitat. Spirurina habitats were significant for genome GC skews, PCG GC skews, dN rates, and dS rates.

Additional file 22: Fig. S15: Spirurina Mitogenome Characteristics by Reproduction. Box and whisker plots for total genome and PCG characteristics for A) size, B) %GC content, C) GC compositional skew, and D) substitution rates for PCG sequences for the Spirurina suborder. Medians and quantiles were calculated for each characteristic based on the life traits classification for Reproduction strategy. Spirurina reproductive strategies were not significant for any characteristics.

## Data Availability

Raw sequences and metadata generated for this study have been deposited into the NCBI Sequence Read Archive (SRA) under BioProject PRJNA852289 with the accession IDs SAMN29272362– SAMN29272480. Assembled and annotated gnomes will be available from NCBI upon acceptance of the manuscript. The pipeline, code, and dataset, including assembled genomes and annotated gene sequences, used to process and analyze the data are available at https://github.com/WormsEtAl/Nematode-Comparative-Mitochondrial-Genomics.
