# Supplementary material for: Comparative mitochondrial genomics in Nematoda reveal astonishing variation in compositional biases and substitution rates indicative of multi-level selection
Source: BMC Genomics. 2024 Jun 18;25:615. doi: 10.1186/s12864-024-10500-1 (PMC11184840; doi:10.1186/s12864-024-10500-1)
Supplement: Supplementary file 7 — Additional file 7: Table S7. Mutational Saturation Statistical Tests. Summary of clade specific tests of mutational saturation for each of the 12 PCGs found in Nematode mitochondrial genomes. Mutational saturation was tested by clade for each PCG (Enoplea, Rhabditina, Tylenchina, and Spirurina). [file 12864_2024_10500_MOESM7_ESM.pdf]

Supplemental Figure 7: Tylenchina Mitogenome Characteristics and Substitution Rates by Habit

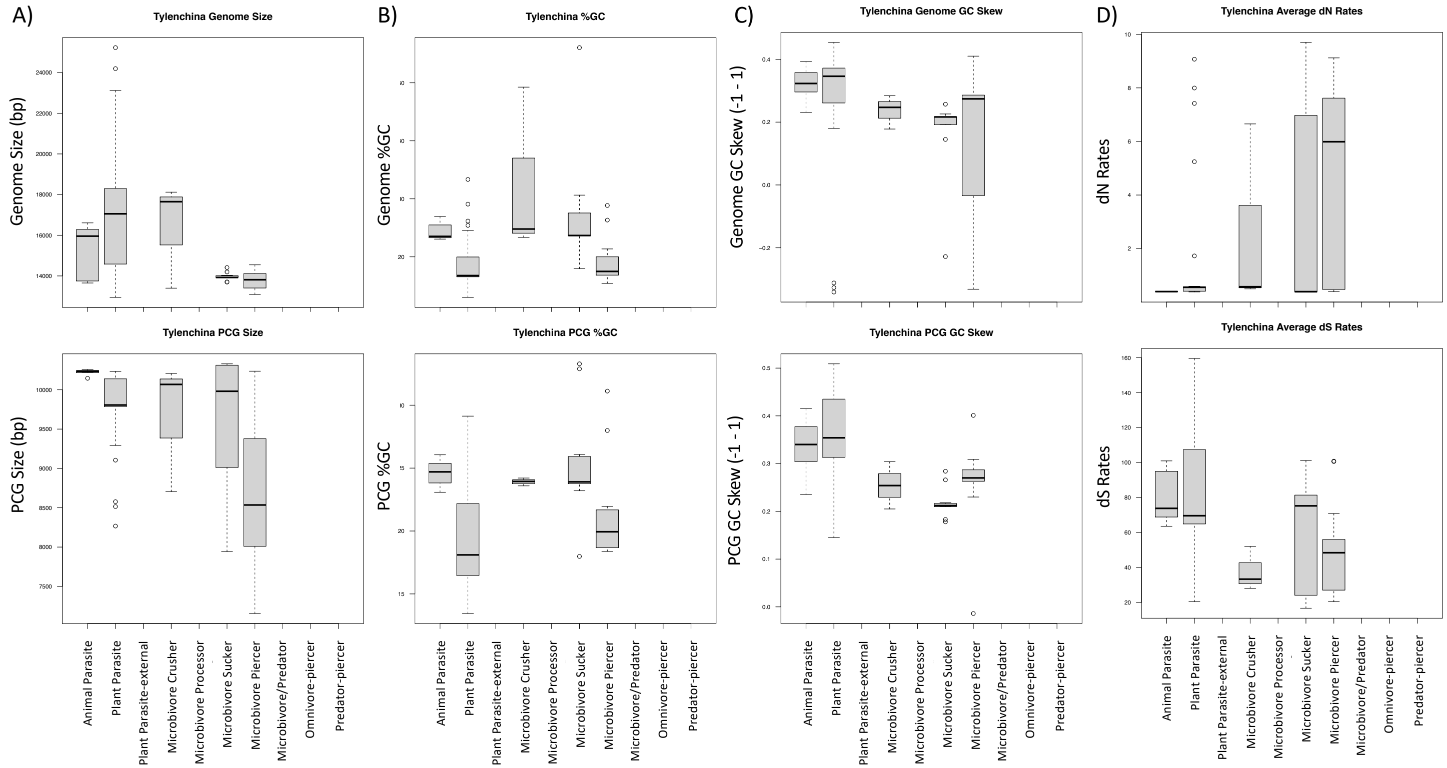

SI Figure 7: Tylenchina Mitogenome Characteristics by Feeding Habit

Box and whisker plots for total genome and PCG characteristics for A) size, B) %GC content, C) GC compositional skew, and D) substitution rates for PCG sequences for the Tylenchina suborder. Medians and quantiles were calculated for each characteristic based on the life trait classification for feeding Habit. Tylenchina feeding habit was significant for genome %GC, Genome GC skews, and PCG GC skews.
