# Supplementary material for: Comparative mitochondrial genomics in Nematoda reveal astonishing variation in compositional biases and substitution rates indicative of multi-level selection
Source: BMC Genomics. 2024 Jun 18;25:615. doi: 10.1186/s12864-024-10500-1 (PMC11184840; doi:10.1186/s12864-024-10500-1)
Supplement: Supplementary file 8 — Additional file 8: Fig. S1: Nematode Mitogenome Characteristics by Feeding Habit. Box and whisker plots for total genome and PCG characteristics for A) size, B) %GC content, C) GC compositional skew, and D) substitution rates for PCG sequences for the Nematoda phylum. Medians and quantiles were calculated for each characteristic based on the life trait classification for feeding Habit. Phylum level feeding habit was significant for all characteristics. [file 12864_2024_10500_MOESM8_ESM.pdf]

Supplemental Figure 1: Nematoda Mitogenome Characteristics and Substitution Rates by Habit

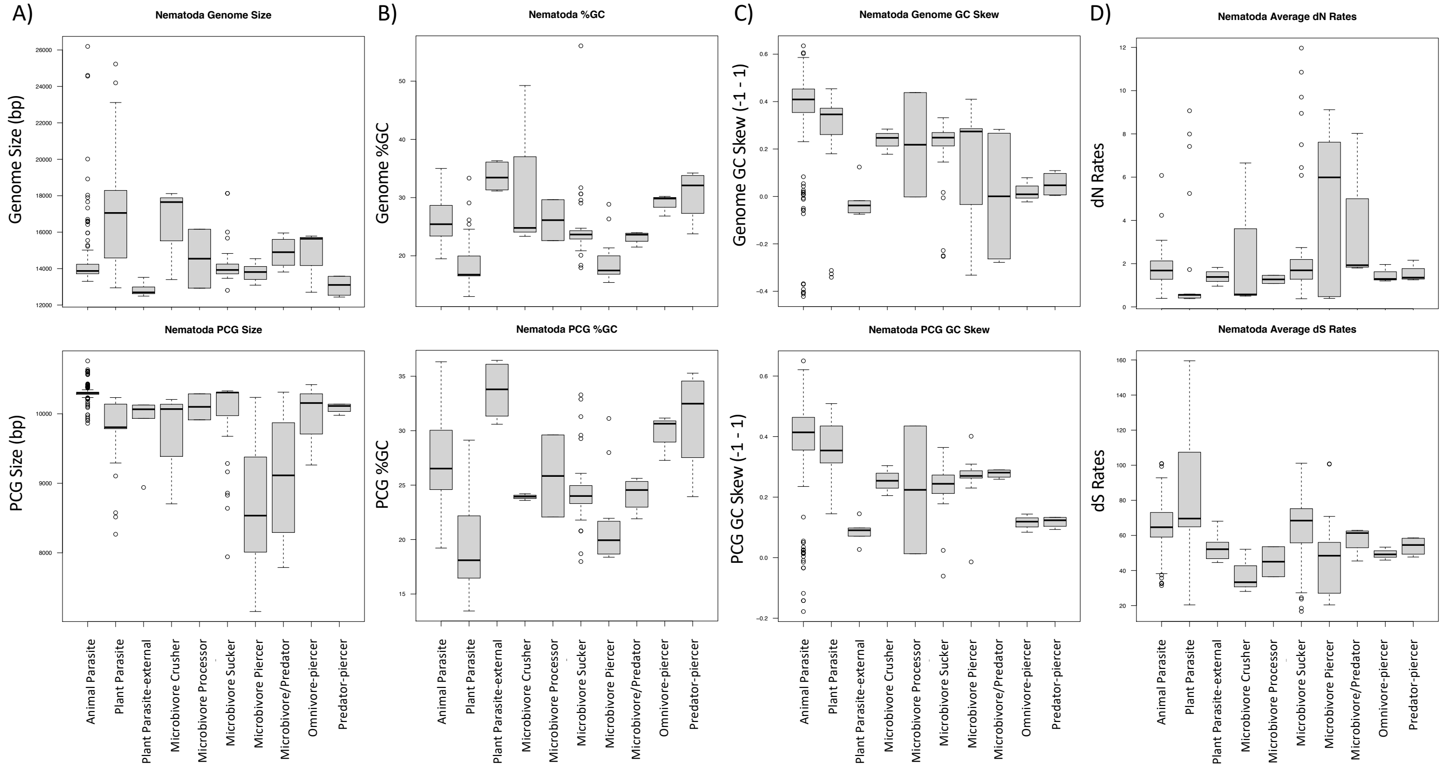

SI Figure 1: Nematode Mitogenome Characteristics by Feeding Habit

Box and whisker plots for total genome and PCG characteristics for A) size, B) %GC content, C) GC compositional skew, and D) substitution rates for PCG sequences for the Nematoda phylum. Medians and quantiles were calculated for each characteristic based on the life trait classification for feeding Habit. Phylum level feeding habit was significant for all characteristics.
