# Supplementary material for: Comparative mitochondrial genomics in Nematoda reveal astonishing variation in compositional biases and substitution rates indicative of multi-level selection
Source: BMC Genomics. 2024 Jun 18;25:615. doi: 10.1186/s12864-024-10500-1 (PMC11184840; doi:10.1186/s12864-024-10500-1)
Supplement: Supplementary file 9 — Additional file 9: Fig. S2: Nematode Mitogenome Characteristics by Habitat. Box and whisker plots for total genome and PCG characteristics for A) size, B) %GC content, C) GC compositional skew, and D) substitution rates for PCG sequences for the Nematoda phylum. Medians and quantiles were calculated for each characteristic based on the life trait classification for preferred Habitat. Phylum level habitat was significant for all characteristics expect dN rates. [file 12864_2024_10500_MOESM9_ESM.pdf]

Supplemental Figure 2: Nematoda Mitogenome Characteristics and Substitution Rates by Habitat

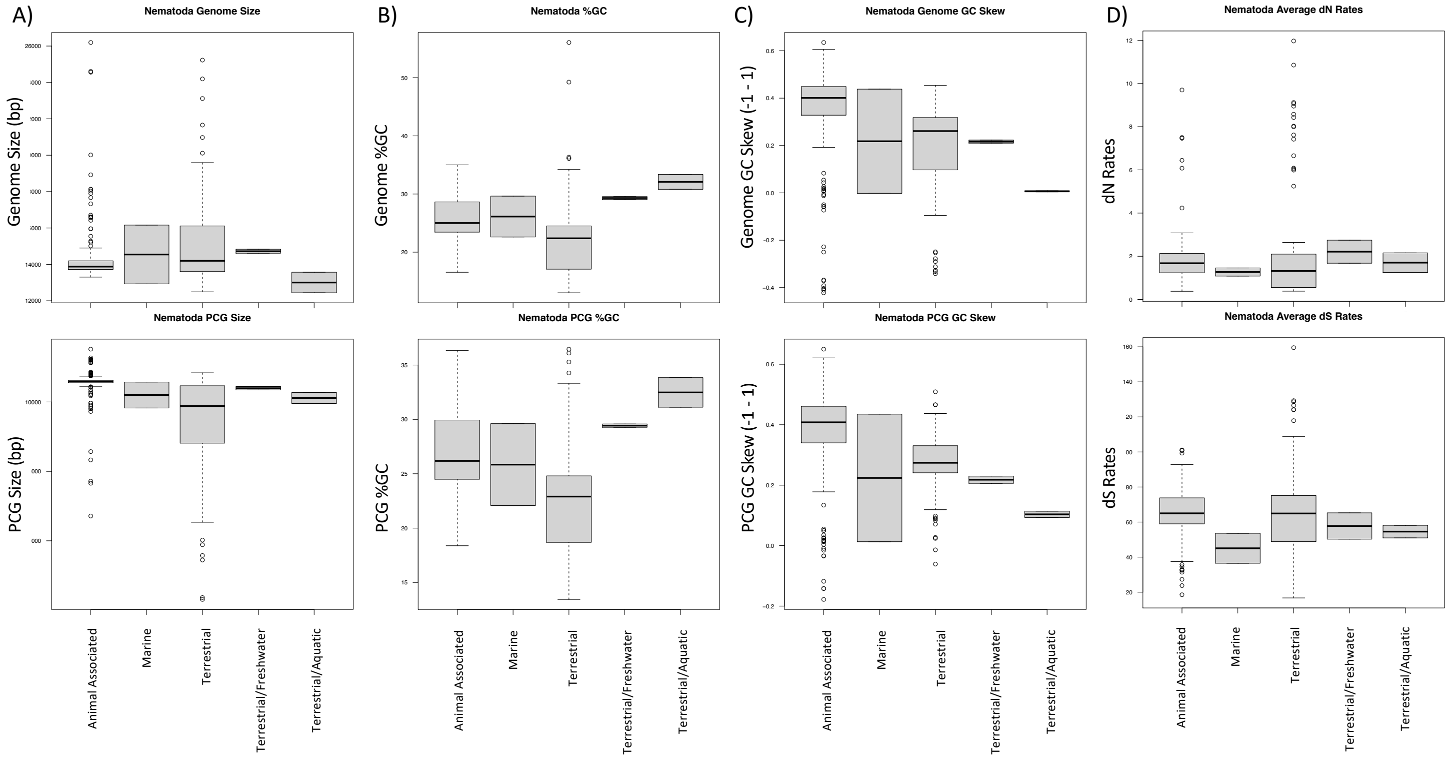

SI Figure 2: Nematode Mitogenome Characteristics by Habitat

Box and whisker plots for total genome and PCG characteristics for A) size, B) %GC content, C) GC compositional skew, and D) substitution rates for PCG sequences for the Nematoda phylum. Medians and quantiles were calculated for each characteristic based on the life trait classification for preferred Habitat. Phylum level habitat was significant for all characteristics expect dN rates.
