# Supplementary material for: Comparative mitochondrial genomics in Nematoda reveal astonishing variation in compositional biases and substitution rates indicative of multi-level selection
Source: BMC Genomics. 2024 Jun 18;25:615. doi: 10.1186/s12864-024-10500-1 (PMC11184840; doi:10.1186/s12864-024-10500-1)
Supplement: Supplementary file 10 — Additional file 10: Fig. S3: Nematode Mitogenome Characteristics by Reproduction. Box and whisker plots for total genome and PCG characteristics for A) size, B) %GC content, C) GC compositional skew, and D) substitution rates for PCG sequences for the Nematoda phylum. Medians and quantiles were calculated for each characteristic based on the life trait classification for Reproduction strategy. Phylum level reproduction strategy was not significant for any characteristic. [file 12864_2024_10500_MOESM10_ESM.pdf]

Supplemental Figure 3: Nematoda Mitogenome Characteristics and Substitution Rates by Reproduction

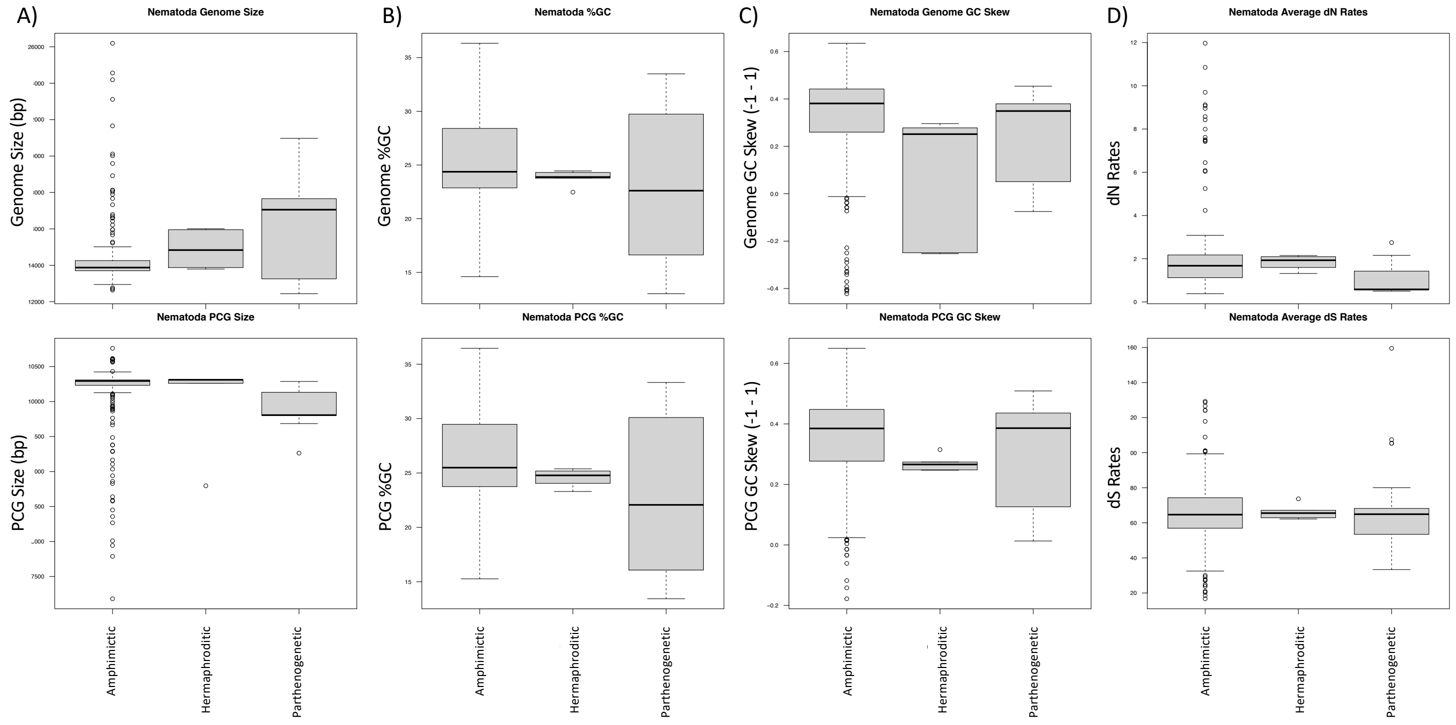

SI Figure 3: Nematode Mitogenome Characteristics by Reproduction

Box and whisker plots for total genome and PCG characteristics for A) size, B) %GC content, C) GC compositional skew, and D) substitution rates for PCG sequences for the Nematoda phylum. Medians and quantiles were calculated for each characteristic based on the life trait classification for Reproduction strategy. Phylum level reproduction strategy was not significant for any characteristic.
