# Supplementary material for: Comparative mitochondrial genomics in Nematoda reveal astonishing variation in compositional biases and substitution rates indicative of multi-level selection
Source: BMC Genomics. 2024 Jun 18;25:615. doi: 10.1186/s12864-024-10500-1 (PMC11184840; doi:10.1186/s12864-024-10500-1)
Supplement: Supplementary file 11 — Additional file 11: Fig. S4: Enoplea Mitogenome Characteristics by Feeding Habit. Box and whisker plots for total genome and PCG characteristics for A) size, B) %GC content, C) GC compositional skew, and D) substitution rates for PCG sequences for the Enoplea class. Medians and quantiles were calculated for each characteristic based on the life trait classification for feeding Habit. Enoplea feed habit was only significant for genome size. [file 12864_2024_10500_MOESM11_ESM.pdf]

Supplemental Figure 4: Enoplea Mitogenome Characteristics and Substitution Rates by Habit

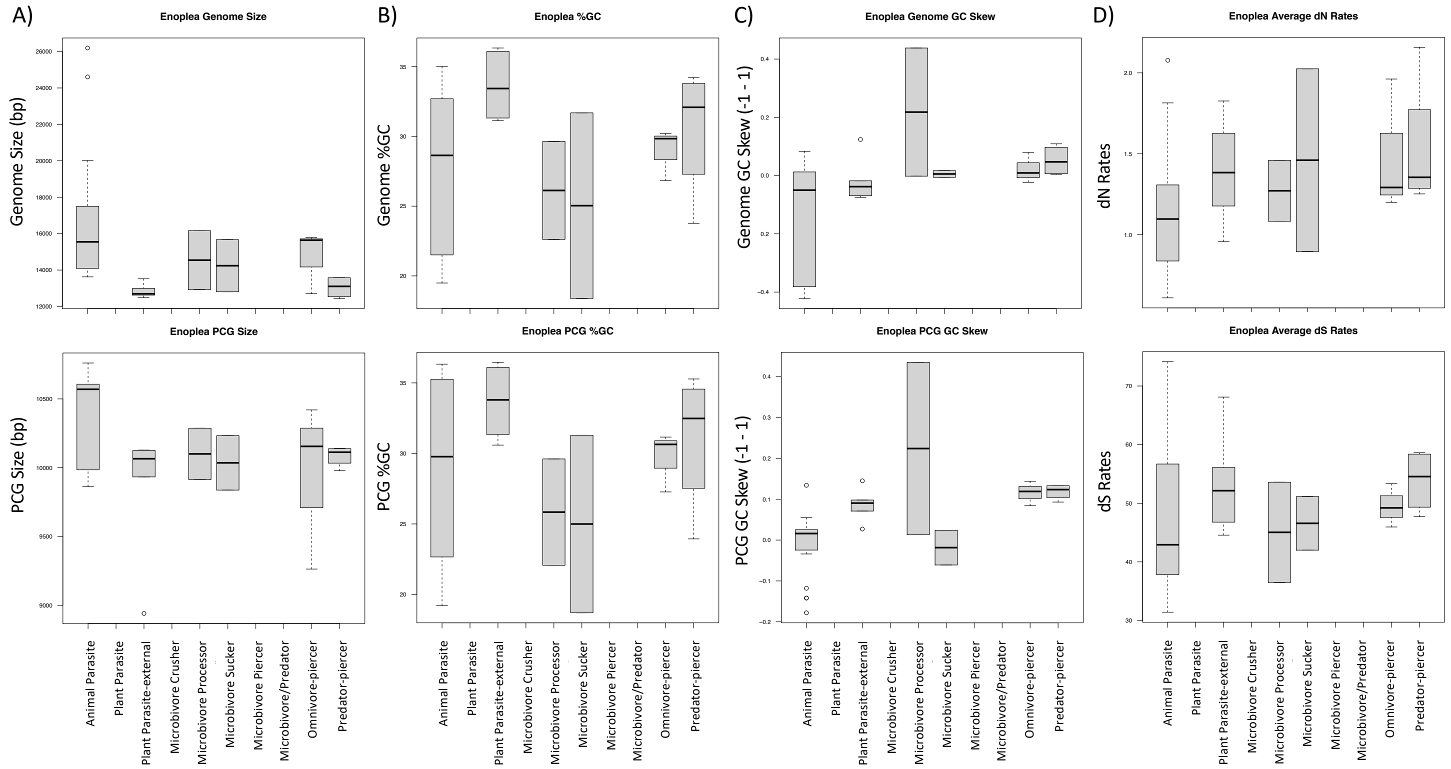

SI Figure 4: Enoplea Mitogenome Characteristics by Feeding Habit

Box and whisker plots for total genome and PCG characteristics for A) size, B) %GC content, C) GC compositional skew, and D) substitution rates for PCG sequences for the Enoplea class. Medians and quantiles were calculated for each characteristic based on the life trait classification for feeding Habit. Enoplea feed habit was only significant for genome size.
