# Supplementary material for: Comparative mitochondrial genomics in Nematoda reveal astonishing variation in compositional biases and substitution rates indicative of multi-level selection
Source: BMC Genomics. 2024 Jun 18;25:615. doi: 10.1186/s12864-024-10500-1 (PMC11184840; doi:10.1186/s12864-024-10500-1)
Supplement: Supplementary file 12 — Additional file 12: Fig. S5: Enoplea Mitogenome Characteristics by Habitat. Box and whisker plots for total genome and PCG characteristics for A) size, B) %GC content, C) GC compositional skew, and D) substitution rates for PCG sequences for the Enoplea class. Medians and quantiles were calculated for each characteristic based on the life trait classification for preferred Habitat. Enoplea habitat was significant for all characteristics expect for dN and dS rates. [file 12864_2024_10500_MOESM12_ESM.pdf]

Supplemental Figure 5: Enoplea Mitogenome Characteristics and Substitution Rates by Habitat

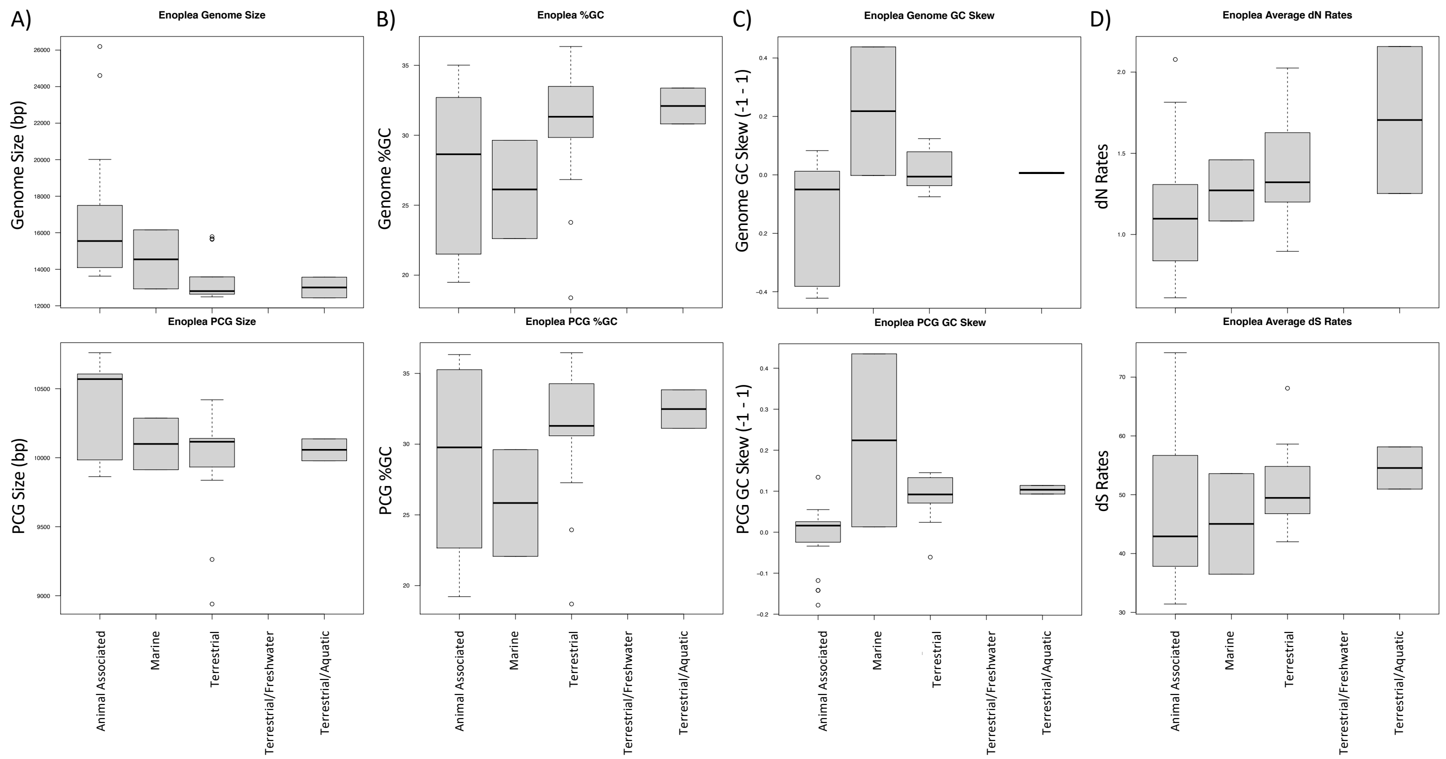

SI Figure 5: Enoplea Mitogenome Characteristics by Habitat

Box and whisker plots for total genome and PCG characteristics for A) size, B) %GC content, C) GC compositional skew, and D) substitution rates for PCG sequences for the Enoplea class. Medians and quantiles were calculated for each characteristic based on the life trait classification for preferred Habitat. Enoplea habitat was significant for all characteristics expect for dN and dS rates.
