# Supplementary material for: Comparative mitochondrial genomics in Nematoda reveal astonishing variation in compositional biases and substitution rates indicative of multi-level selection
Source: BMC Genomics. 2024 Jun 18;25:615. doi: 10.1186/s12864-024-10500-1 (PMC11184840; doi:10.1186/s12864-024-10500-1)
Supplement: Supplementary file 13 — Additional file 13: Fig. S6: Enoplea Mitogenome Characteristics by Reproduction. Box and whisker plots for total genome and PCG characteristics for A) size, B) %GC content, C) GC compositional skew, and D) substitution rates for PCG sequences for the Enoplea class. Medians and quantiles were calculated for each characteristic based on the life trait classification for Reproduction strategy. Enoplea reproduction strategy was only significant for genome size. [file 12864_2024_10500_MOESM13_ESM.pdf]

Supplemental Figure 6: Enoplea Mitogenome Characteristics and Substitution Rates by Reproduction

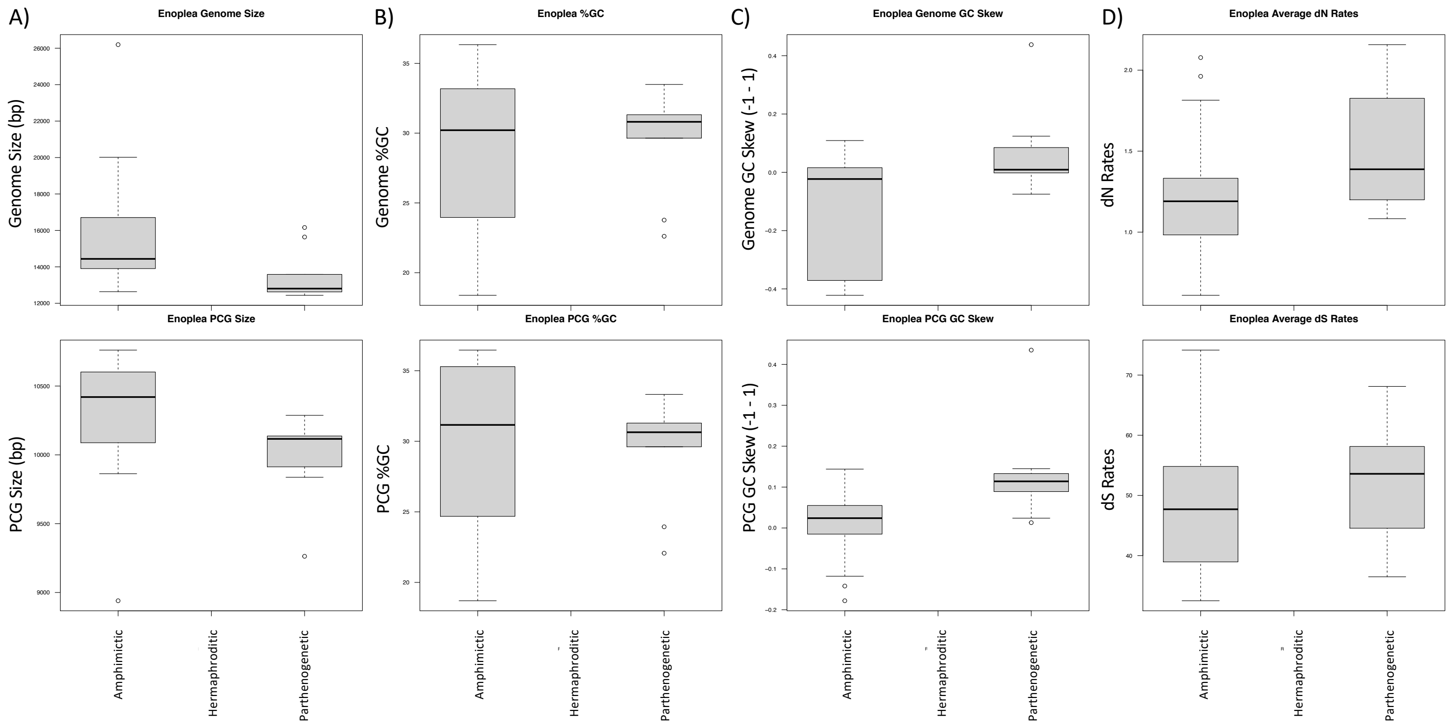

SI Figure 6: Enoplea Mitogenome Characteristics by Reproduction

Box and whisker plots for total genome and PCG characteristics for A) size, B) %GC content, C) GC compositional skew, and D) substitution rates for PCG sequences for the Enoplea class. Medians and quantiles were calculated for each characteristic based on the life trait classification for Reproduction strategy. Enoplea reproduction strategy was only significant for genome size.
