# Supplementary material for: Comparative mitochondrial genomics in Nematoda reveal astonishing variation in compositional biases and substitution rates indicative of multi-level selection
Source: BMC Genomics. 2024 Jun 18;25:615. doi: 10.1186/s12864-024-10500-1 (PMC11184840; doi:10.1186/s12864-024-10500-1)
Supplement: Supplementary file 15 — Additional file 15: Fig. S8: Tylenchina Mitogenome Characteristics by Habitat. Box and whisker plots for total genome and PCG characteristics for A) size, B) %GC content, C) GC compositional skew, and D) substitution rates for PCG sequences for the Tylenchina suborder. Medians and quantiles were calculated for each characteristic based on the life trait classification for preferred Habitat. Tylenchina habitat was significant for genome %GC, total PCG size, PCG proportion of the genome, PCG GC Skews, and dN rates. [file 12864_2024_10500_MOESM15_ESM.pdf]

Supplemental Figure 8: Tylenchina Mitogenome Characteristics and Substitution Rates by Habitat

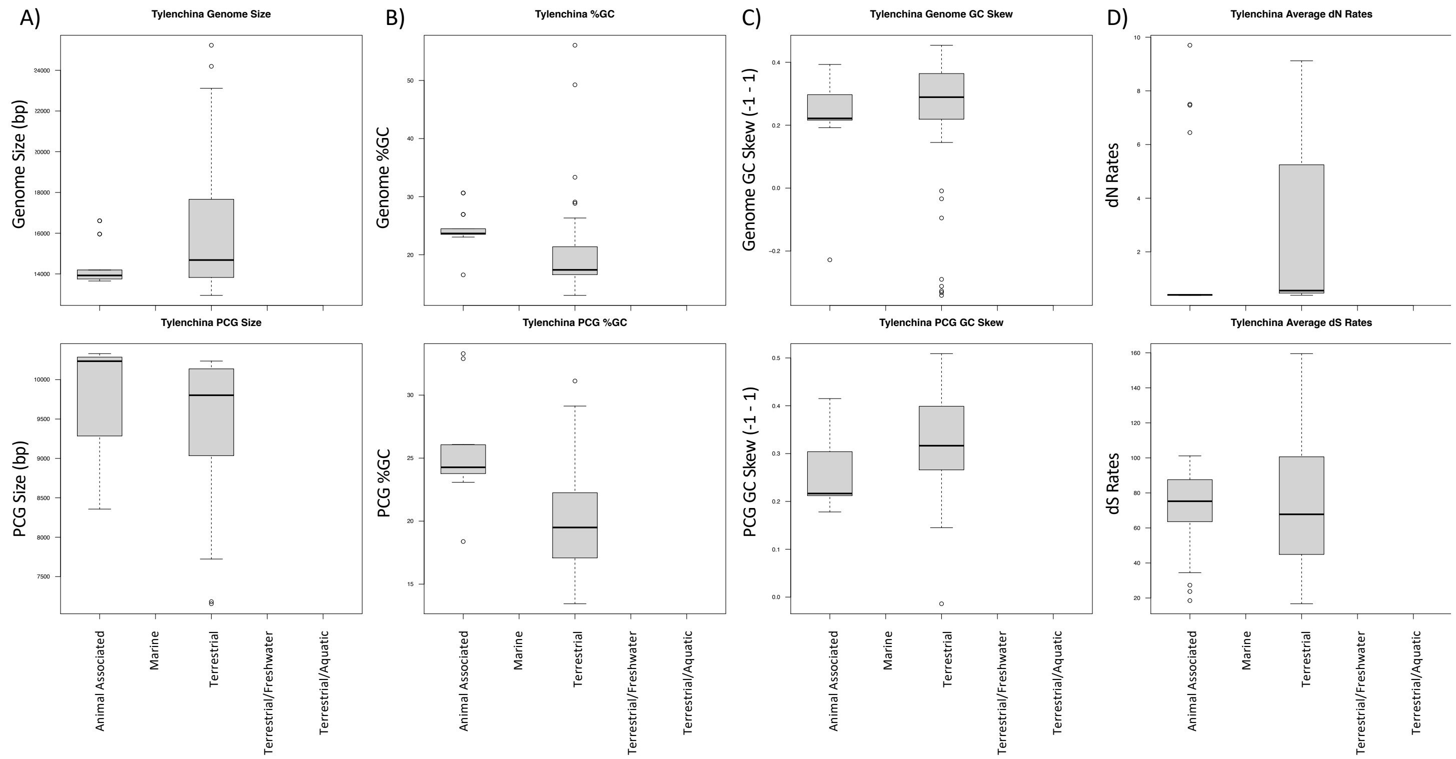

SI Figure 8: Tylenchina Mitogenome Characteristics by Habitat

Box and whisker plots for total genome and PCG characteristics for A) size, B) %GC content, C) GC compositional skew, and D) substitution rates for PCG sequences for the Tylenchina suborder. Medians and quantiles were calculated for each characteristic based on the life trait classification for preferred Habitat. Tylenchina habitat was significant for genome %GC, total PCG size, PCG proportion of the genome, PCG GC Skews, and dN rates.
