# Supplementary material for: Comparative mitochondrial genomics in Nematoda reveal astonishing variation in compositional biases and substitution rates indicative of multi-level selection
Source: BMC Genomics. 2024 Jun 18;25:615. doi: 10.1186/s12864-024-10500-1 (PMC11184840; doi:10.1186/s12864-024-10500-1)
Supplement: Supplementary file 16 — Additional file 16: Fig. S9: Tylenchina Mitogenome Characteristics by Reproduction. Box and whisker plots for total genome and PCG characteristics for A) size, B) %GC content, C) GC compositional skew, and D) substitution rates for PCG sequences for the Tylenchina suborder. Medians and quantiles were calculated for each characteristic based on the life trait classification for Reproduction strategy. Tylenchina reproduction strategy was significant for genome size, genome %GC, PCG proportion of the genome, Genome GC skews, and PCG GC skews. [file 12864_2024_10500_MOESM16_ESM.pdf]

Supplemental Figure 9: Tylenchina Mitogenome Characteristics and Substitution Rates by Reproduction

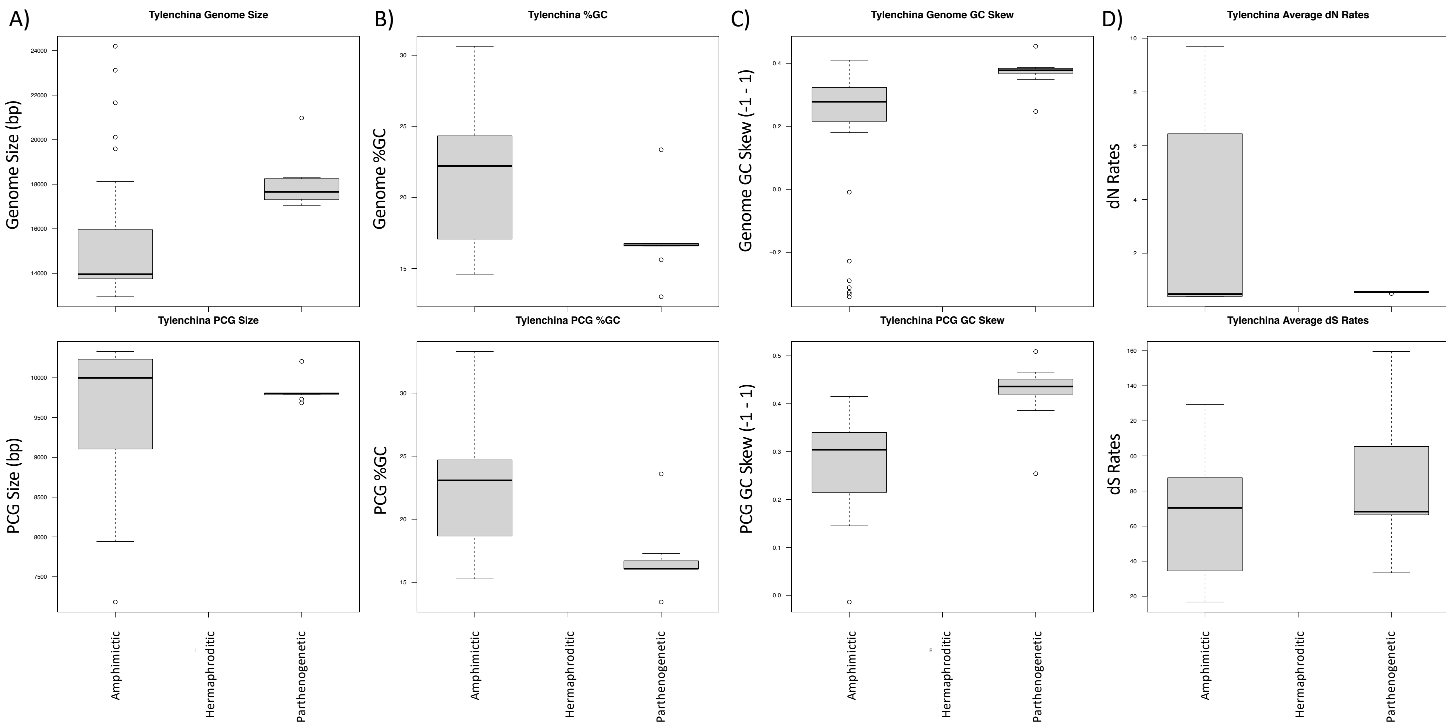

SI Figure 9: Tylenchina Mitogenome Characteristics by Reproduction

Box and whisker plots for total genome and PCG characteristics for A) size, B) %GC content, C) GC compositional skew, and D) substitution rates for PCG sequences for the Tylenchina suborder. Medians and quantiles were calculated for each characteristic based on the life trait classification for Reproduction strategy. Tylenchina reproduction strategy was significant for genome size, genome %GC, PCG proportion of the genome, Genome GC skews, and PCG GC skews.
