# Supplementary material for: Comparative mitochondrial genomics in Nematoda reveal astonishing variation in compositional biases and substitution rates indicative of multi-level selection
Source: BMC Genomics. 2024 Jun 18;25:615. doi: 10.1186/s12864-024-10500-1 (PMC11184840; doi:10.1186/s12864-024-10500-1)
Supplement: Supplementary file 17 — Additional file 17: Fig. S10: Rhabditina Mitogenome Characteristics by Feeding Habit. Box and whisker plots for total genome and PCG characteristics for A) size, B) %GC content, C) GC compositional skew, and D) substitution rates for PCG sequences for the Rhabditina suborder. Medians and quantiles were calculated for each characteristic based on the life trait classification for feeding Habit. Rhabditina feeding habits were significant for PCG proportion of the genome, genome GC skews, and PCG GC skews. [file 12864_2024_10500_MOESM17_ESM.pdf]

Supplemental Figure 10: Rhabditina Mitogenome Characteristics and Substitution Rates by Habit

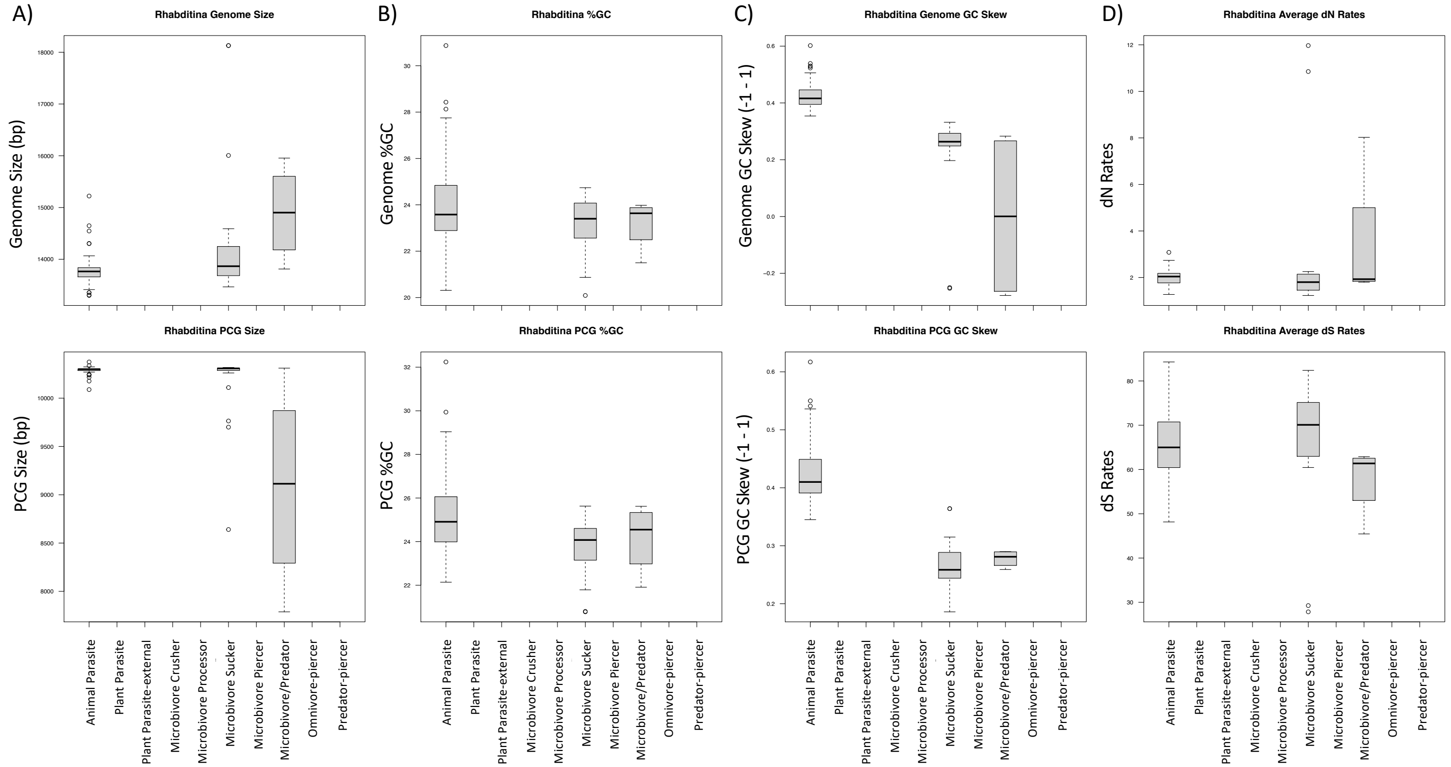

SI Figure 10: Rhabditina Mitogenome Characteristics by Feeding Habit

Box and whisker plots for total genome and PCG characteristics for A) size, B) %GC content, C) GC compositional skew, and D) substitution rates for PCG sequences for the Rhabditina suborder. Medians and quantiles were calculated for each characteristic based on the life trait classification for feeding Habit. Rhabditina feeding habits were significant for PCG proportion of the genome, genome GC skews, and PCG GC skews.
