# Supplementary material for: Comparative mitochondrial genomics in Nematoda reveal astonishing variation in compositional biases and substitution rates indicative of multi-level selection
Source: BMC Genomics. 2024 Jun 18;25:615. doi: 10.1186/s12864-024-10500-1 (PMC11184840; doi:10.1186/s12864-024-10500-1)
Supplement: Supplementary file 18 — Additional file 18: Fig. S11: Rhabditina Mitogenome Characteristics by Habitat. Box and whisker plots for total genome and PCG characteristics for A) size, B) %GC content, C) GC compositional skew, and D) substitution rates for PCG sequences for the Rhabditina suborder. Medians and quantiles were calculated for each characteristic based on the life trait classification for preferred Habitat. Rhabditina habitats were significant for PCG proportion of the genome, genome GC skews, and PCG GC skews. [file 12864_2024_10500_MOESM18_ESM.pdf]

Supplemental Figure 11: Rhabditina Mitogenome Characteristics and Substitution Rates by Habitat

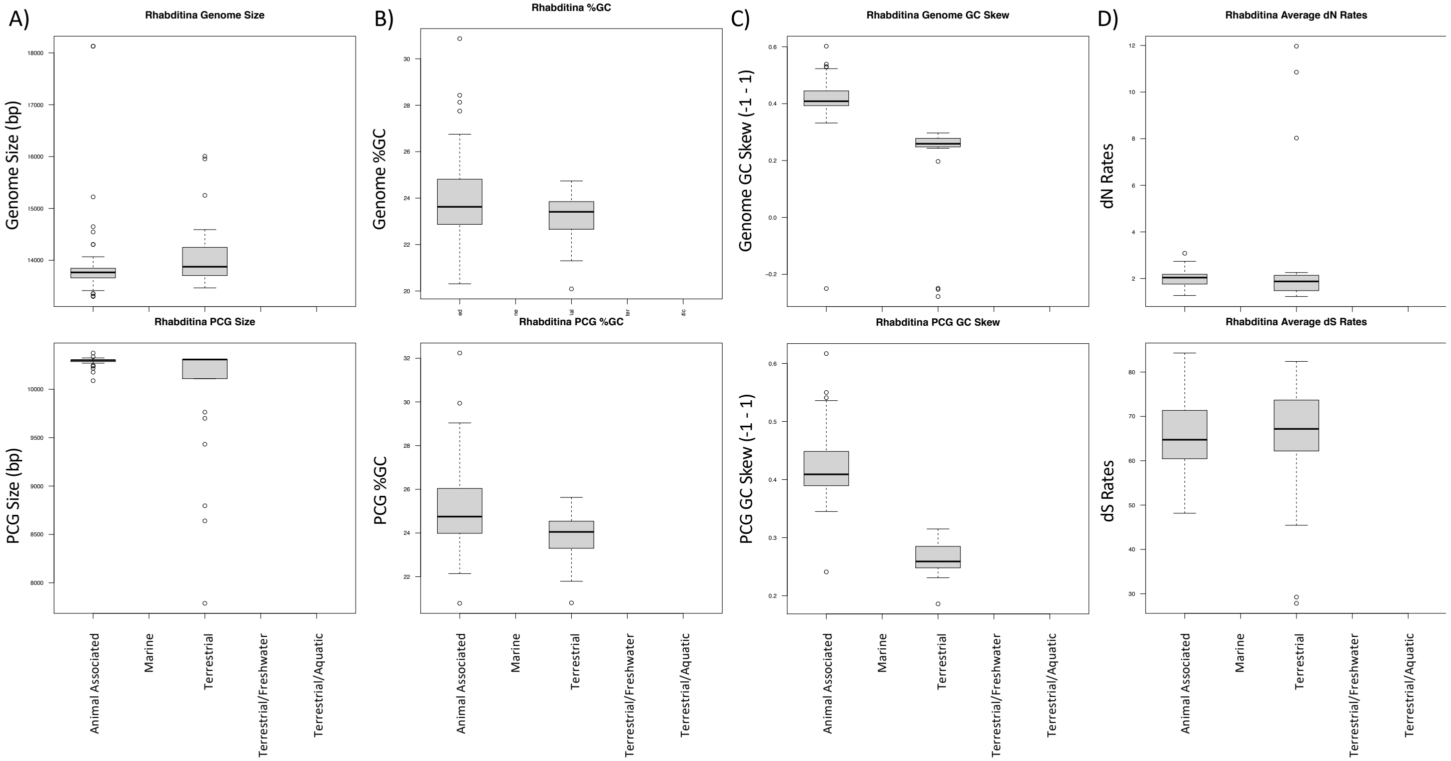

SI Figure 11: Rhabditina Mitogenome Characteristics by Habitat

Box and whisker plots for total genome and PCG characteristics for A) size, B) %GC content, C) GC compositional skew, and D) substitution rates for PCG sequences for the Rhabditina suborder. Medians and quantiles were calculated for each characteristic based on the life trait classification for preferred Habitat. Rhabditina habitats were significant for PCG proportion of the genome, genome GC skews, and PCG GC skews.
