# Supplementary material for: Comparative mitochondrial genomics in Nematoda reveal astonishing variation in compositional biases and substitution rates indicative of multi-level selection
Source: BMC Genomics. 2024 Jun 18;25:615. doi: 10.1186/s12864-024-10500-1 (PMC11184840; doi:10.1186/s12864-024-10500-1)
Supplement: Supplementary file 20 — Additional file 20: Fig. S13: Spirurina Mitogenome Characteristics by Feeding Habit. Box and whisker plots for total genome and PCG characteristics for A) size, B) %GC content, C) GC compositional skew, and D) substitution rates for PCG sequences for the Spirurina suborder. Medians and quantiles were calculated for each characteristic based on the life traits classification for feeding Habit. Spirurina feeding habits were not significant for any characteristics. [file 12864_2024_10500_MOESM20_ESM.pdf]

Supplemental Figure 13: Spirurina Mitogenome Characteristics and Substitution Rates by Habit

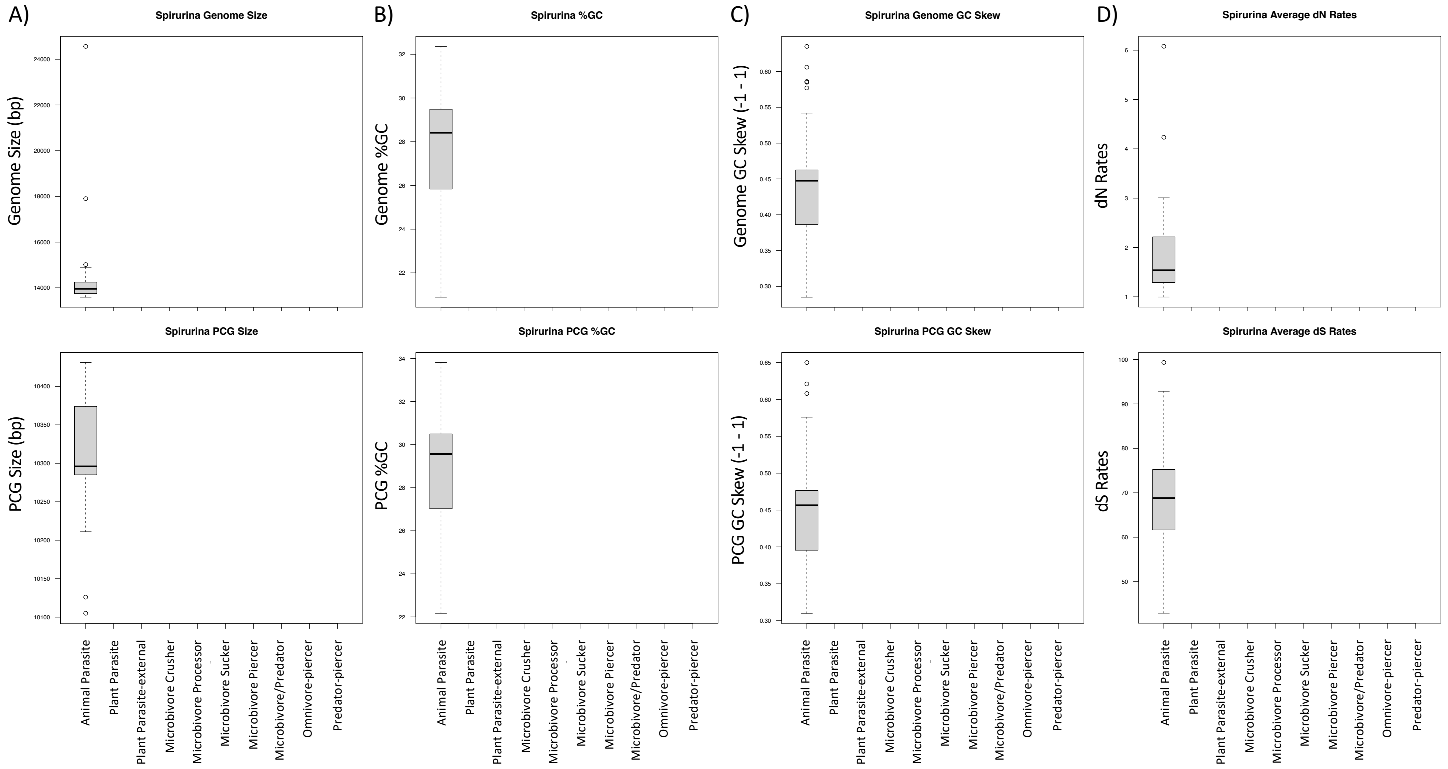

SI Figure 13: Spirurina Mitogenome Characteristics by Feeding Habit

Box and whisker plots for total genome and PCG characteristics for A) size, B) %GC content, C) GC compositional skew, and D) substitution rates for PCG sequences for the Spirurina suborder. Medians and quantiles were calculated for each characteristic based on the life traits classification for feeding Habit. Spirurina feeding habits were not significant for any characteristics.
