# Supplementary material for: Comparative mitochondrial genomics in Nematoda reveal astonishing variation in compositional biases and substitution rates indicative of multi-level selection
Source: BMC Genomics. 2024 Jun 18;25:615. doi: 10.1186/s12864-024-10500-1 (PMC11184840; doi:10.1186/s12864-024-10500-1)
Supplement: Supplementary file 21 — Additional file 21: Fig. S14: Spirurina Mitogenome Characteristics by Habitat. Box and whisker plots for total genome and PCG characteristics for A) size, B) %GC content, C) GC compositional skew, and D) substitution rates for PCG sequences for the Spirurina suborder. Medians and quantiles were calculated for each characteristic based on the life traits classification for preferred Habitat. Spirurina habitats were significant for genome GC skews, PCG GC skews, dN rates, and dS rates. [file 12864_2024_10500_MOESM21_ESM.pdf]

Supplemental Figure 14: Spirurina Mitogenome Characteristics and Substitution Rates by Habitat

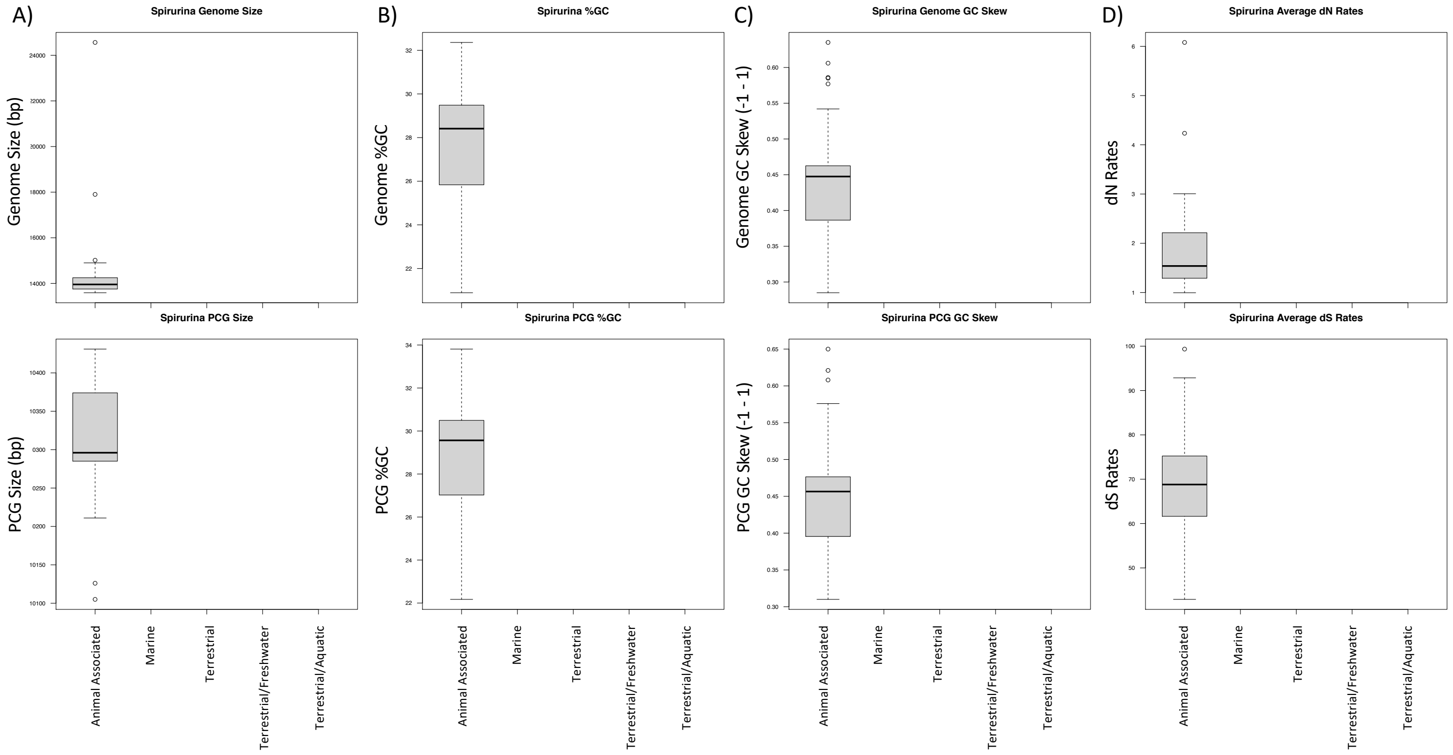

SI Figure 14: Spirurina Mitogenome Characteristics by Habitat

Box and whisker plots for total genome and PCG characteristics for A) size, B) %GC content, C) GC compositional skew, and D) substitution rates for PCG sequences for the Spirurina suborder. Medians and quantiles were calculated for each characteristic based on the life traits classification for preferred Habitat. Spirurina habitats were significant for genome GC skews, PCG GC skews, dN rates, and dS rates.
