# Supplementary material for: Comparative mitochondrial genomics in Nematoda reveal astonishing variation in compositional biases and substitution rates indicative of multi-level selection
Source: BMC Genomics. 2024 Jun 18;25:615. doi: 10.1186/s12864-024-10500-1 (PMC11184840; doi:10.1186/s12864-024-10500-1)
Supplement: Supplementary file 22 — Additional file 22: Fig. S15: Spirurina Mitogenome Characteristics by Reproduction. Box and whisker plots for total genome and PCG characteristics for A) size, B) %GC content, C) GC compositional skew, and D) substitution rates for PCG sequences for the Spirurina suborder. Medians and quantiles were calculated for each characteristic based on the life traits classification for Reproduction strategy. Spirurina reproductive strategies were not significant for any characteristics. [file 12864_2024_10500_MOESM22_ESM.pdf]

Supplemental Figure 15: Spirurina Mitogenome Characteristics and Substitution Rates by Reproduction

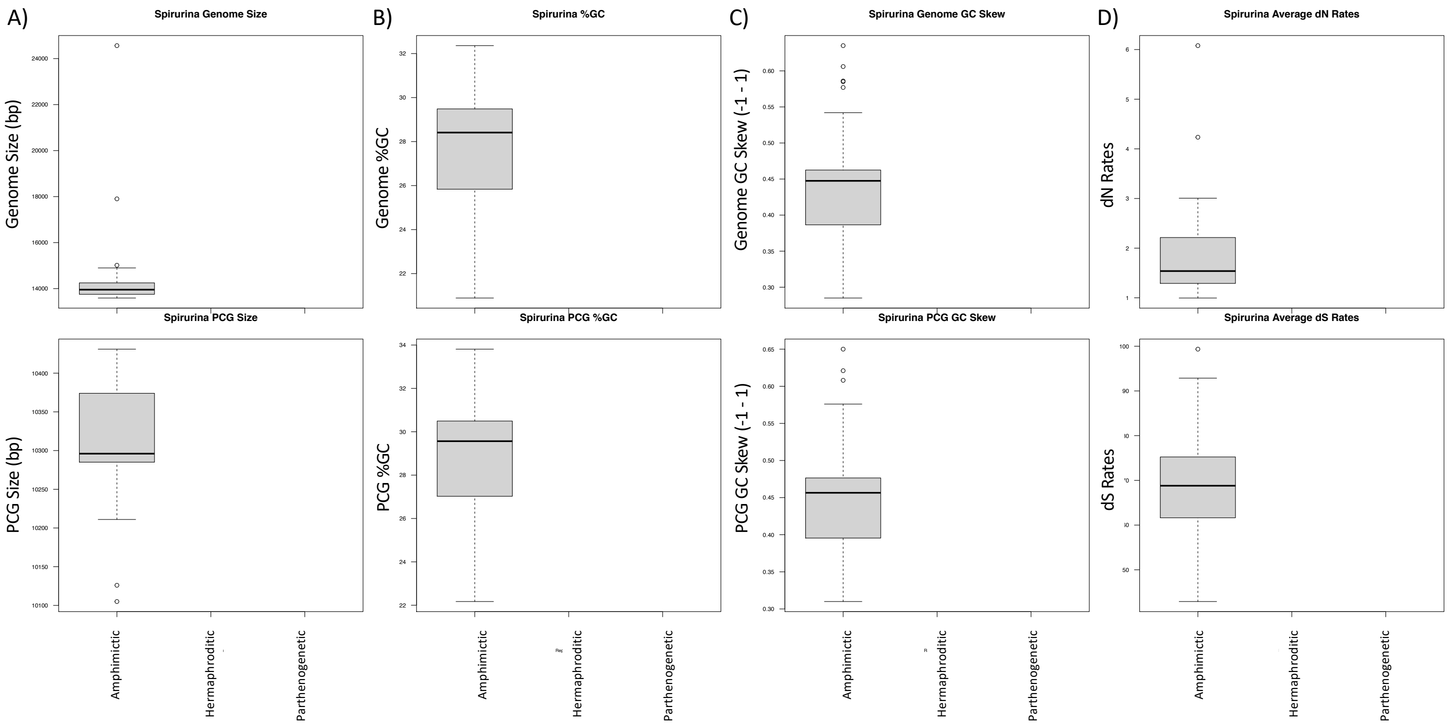

SI Figure 15: Spirurina Mitogenome Characteristics by Reproduction

Box and whisker plots for total genome and PCG characteristics for A) size, B) %GC content, C) GC compositional skew, and D) substitution rates for PCG sequences for the Spirurina suborder. Medians and quantiles were calculated for each characteristic based on the life traits classification for Reproduction strategy. Spirurina reproductive strategies were not significant for any characteristics.
